# Hydrogel Network Architecture Design Space: Impact on Mechanical and Viscoelastic Properties

**DOI:** 10.3390/gels11080588

**Published:** 2025-07-30

**Authors:** Andres F. Roca-Arroyo, Jhonatan A. Gutierrez-Rivera, Logan D. Morton, David A. Castilla-Casadiego

**Affiliations:** 1 Department of Biomedical Engineering, University of Miami, Coral Gables, FL 33146, USA; afr130@miami.edu (A.F.R.-A.); jag932@miami.edu (J.A.G.-R.); 2 Department of Biomedical Engineering, Tufts University, Medford, MA 02155, USA

**Keywords:** hydrogel, crosslinker architecture, viscoelasticity, biomimetic, structure–property relationships, extracellular matrix mimic

## Abstract

This comprehensive review explores the expansive design space of network architectures and their significant impact on the mechanical and viscoelastic properties of hydrogel systems. By examining the intricate relationships between molecular structure, network connectivity, and resulting bulk properties, we provide critical insights into rational design strategies for tailoring hydrogel mechanics for specific applications. Recent advances in sequence-defined crosslinkers, dynamic covalent chemistries, and biomimetic approaches have significantly expanded the toolbox for creating hydrogels with precisely controlled viscoelasticity, stiffness, and stress relaxation behavior—properties that are crucial for biomedical applications, particularly in tissue engineering and regenerative medicine.

## 1. Introduction

Hydrogels represent a class of soft materials characterized by three-dimensional networks of hydrophilic polymers capable of absorbing substantial amounts of water (typically exceeding 90% by weight) while maintaining structural integrity. The unique combination of solid-like mechanical properties and high water content makes hydrogels particularly attractive for a wide range of applications, from biomedical implants and drug delivery systems to tissue engineering scaffolds and soft robotics [1]. The mechanical behavior of these materials, encompassing stiffness, viscoelasticity, and toughness, is predominantly governed by the molecular architecture of the polymer network [2].

Crosslinkers, the molecular entities responsible for connecting polymer chains into cohesive networks, constitute a rapidly developing design space that offers tremendous potential for tuning hydrogel mechanics [3]. The chemical composition, molecular weight, architecture, valency, and dynamic properties of crosslinks collectively create a multidimensional parameter space that can be strategically exploited to engineer hydrogels with precisely defined mechanical characteristics. Understanding the underlying design rules between crosslinker architecture and resultant network properties is essential for developing next-generation materials with enhanced performance and functionality.

The concept of hydrogels dates back to 1894, when the term was first used to describe colloids made of inorganic salts [4,5]. However, the field remained relatively unexplored until the mid-20th century, when the first biomedical hydrogel, Ivalon (poly(vinyl alcohol) crosslinked with formaldehyde) was reported in 1949 [6]. A pivotal moment in hydrogel development occurred in 1960 when Wichterle and Lim pioneered the use of poly(2-hydroxymethyl methacrylate) (pHEMA) gels for soft contact lenses, marking the beginning of hydrogels for biological applications [7]. Shortly thereafter, poly(vinyl alcohol) (PVA) hydrogels and polyacrylamide (PAAm) networks also emerged for biomedical uses, including as implants and wound dressings, illustrating the rapid diversification of hydrogel systems for healthcare.

Concurrently, fundamental understanding of polymer network formation was progressing significantly, with seminal contributions from Flory and Stockmayer in the 1940s establishing the theoretical framework for step-growth polymerization networks [8,9,10]. These theoretical foundations, combined with experimental advances, have enabled increasingly sophisticated approaches to controlling network architecture.

Mechanical properties (stiffness, toughness, etc.) are often the most important consideration for hydrogel utilization in any application [11]. In biomedical contexts, mechanics have been repeatedly shown to influence and even direct cellular behavior [12]. Cells are exquisitely sensitive to their mechanical microenvironment, employing sophisticated mechanotransduction pathways to sense and respond to substrate properties [13]. Consequently, the stiffness, viscoelasticity, and stress relaxation characteristics of hydrogels directly modulate critical cellular processes including adhesion, spreading, migration, proliferation, and differentiation [14].

Beyond biomedical applications, which make up the majority of recent work in this space, mechanical properties determine performance in soft robotics [15], actuators [16], sensors [17], and pharmaceuticals [18]. The ability to precisely engineer mechanical responses enables application-specific material design.

Despite significant advances in our understanding of hydrogels, fabricating materials with precisely controlled mechanical properties remains challenging. Conventional approaches to modulating hydrogel mechanics have focused on adjusting polymer concentration, molecular weight, or crosslinking density. While these methods are well documented and reliably modulate stiffness, they simultaneously affect swelling, porosity, ligand density, degradation (if the crosslinks are degradable), and diffusivity [19]. This interdependence limits independent control over specific mechanical characteristics.

Creating homogeneous networks with uniform crosslinking distribution represents another significant challenge. Heterogeneities in crosslinking density introduce local variations in mechanical properties, potentially compromising overall material performance [20]. These effects are particularly pronounced at high strains, where network defects and inhomogeneities dramatically influence mechanical behavior [21].

The complex relationship between molecular scale crosslinker design and macroscale mechanical properties further complicates rational material engineering. Multiple hierarchical levels, from individual chemical bonds to supramolecular assemblies to bulk material properties, must be considered simultaneously. Moreover, predicting how network architecture influences dynamic properties such as stress relaxation, creep, and self-healing requires an additional layer of time-dependent analysis. This is typically accomplished through rheological approaches, including oscillatory shear measurements, stress–relaxation tests, and creep-recovery experiments, which together provide critical insights into the viscoelastic landscape of hydrogel systems.

The native extracellular matrix (ECM) exhibits complex viscoelastic behavior, with tissue-specific mechanical signatures that evolve dynamically during development, homeostasis, and disease progression [22]. Recapitulating these nuanced mechanical properties is essential for creating biomimetic hydrogels capable of directing cell fate and tissue formation. Strain–stiffening behavior, characteristic of many biological tissues, provides a protective mechanism against mechanical damage while enabling mechanically responsive cellular microenvironments—a feature increasingly recognized as critical for successful tissue engineering strategies [23]. For instance, fibrous collagen networks uncrimp and align under tension, increasing stiffness at high strain to prevent damage, then returning to a relaxed state when the stress is removed [24]. The ECM’s time-dependent mechanical properties (viscoelasticity, adaptive stiffening, gradual stress relaxation) play a pivotal role in regulating cellular processes like adhesion, migration, and differentiation [25]. To further complicate this picture, cells constantly remodel the ECM, secreting enzymes that break down crosslinks and proteins that form new ones to sculpt their surrounding microenvironment. Attempts to mimic this complexity have resulted in incorporating multiple types of crosslinks, hierarchical structures, and stimuli-responsive mechanics [22]. Accordingly, the primary aim of this review is to systematically elucidate how variations in crosslinker architecture (including their chemical composition, topology, and dynamic bonding mechanisms) govern the mechanical and viscoelastic properties of hydrogel systems. By establishing clear structure–property relationships, we seek to provide guiding principles for the rational design of hydrogels with tailored mechanics and dynamic behavior, to meet the diverse demands of biomedical and soft-material applications.

## 2. Crosslinker Architecture Design Space

**Figure 1:**
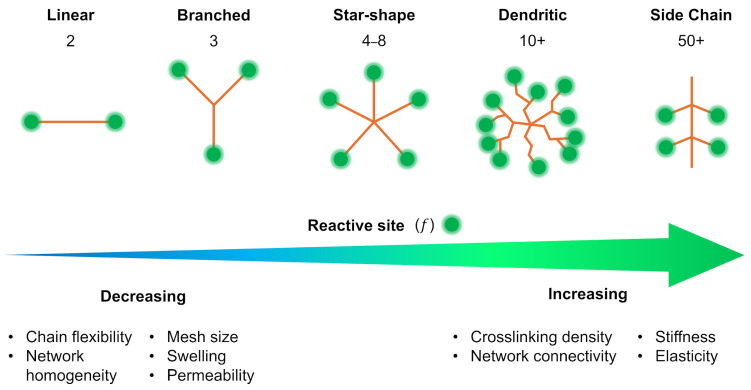
Schematic representation of crosslinker architectures highlighting the progression from linear (difunctional) to increasingly complex topologies such as branched, star-shaped, dendritic, and side-chain structures. The number of reactive sites (*f*) increases across this spectrum, leading to higher crosslinking density, network connectivity, stiffness, and elasticity, while simultaneously reducing chain flexibility, mesh size, swelling, permeability, and network homogeneity. This structural continuum underscores how crosslinker design serves as a versatile handle for tuning hydrogel mechanics and transport properties.

The architecture of a crosslinker (its spatial structure, chemistry, topology, and connectivity) defines a critical design space for tuning the physical and mechanical properties of hydrogels. Beyond simple linear or difunctional linkers, more complex architectures such as branched, star-shaped, and dendritic crosslinkers enable precise control over network density, elasticity, and porosity (Figure 1). These architectural variations influence how stress is distributed throughout the network, how degradation or remodeling occurs, and how diffusion is regulated. For example, star-shaped crosslinkers can create more uniformly distributed junctions and enhance network stability [26], while flexible or asymmetrical architectures can introduce viscoelastic behavior or responsiveness to external stimuli [27]. By engineering network architecture in concert with chemical composition, researchers can access a highly tunable material design space capable of meeting diverse biomedical and engineering demands. See Table 1 for a complete list of the crosslinking systems discussed.

### 2.1. Chemical Composition of Crosslinkers

The chemical composition of a crosslinker does far more than simply “lock” polymer chains together—it dictates nearly every aspect of how the resulting hydrogel will behave in vitro and in vivo. The reactive moieties (e.g., acrylates, maleimides, azides/alkynes for click chemistry, or enzyme-cleavable peptide handles) govern the efficiency and orthogonality of network formation, which in turn affects gelation time and network homogeneity. Incorporating flexible crosslinkers such as polyethylene glycol (PEG) yields highly elastic, water-rich networks, whereas aromatic or urethane-containing crosslinkers introduce rigidity and higher modulus.

Beyond mechanics, chemistry determines how a hydrogel interacts with biological systems. Ester- or disulfide-containing crosslinkers promote hydrolytic or reductive degradation, enabling tunable resorption rates, while peptide sequences recognized by matrix metalloproteinases allow cell-mediated remodeling and migration. Moreover, embedding bioactive motifs (e.g., RGD peptides for integrin binding, heparin-mimetic sulfates for growth-factor sequestration, or catechols for wet adhesion) confers specific cell-instructive or functional properties. Because these molecular building blocks can be mixed and matched in a modular fashion, researchers can systematically design hydrogels whose mechanics, degradation kinetics, and biochemical signaling are co-optimized for applications ranging from injectable tissue scaffolds and immunomodulatory drug depots to soft actuators and 3D bioprinting inks.

#### 2.1.1. Synthetic Crosslinkers

Synthetic polymer crosslinkers are man-made molecules designed with reactive groups that can form bridges between polymer network chains. The prototypical example is PEG: a neutral, hydrophilic polymer available in various architectures (linear, 4-arm, 8-arm, etc.) and easily functionalized with reactive end-groups (e.g., acrylates, thiols, azides) [52]. PEG-based crosslinkers are widely used due to their biocompatibility, chemical versatility, and lack of biological activity, which allow a hydrogel’s mechanical properties to be tuned independently of its biochemical properties. For instance, PEG diacrylate (PEGDA), a linear difunctional crosslinker, is commonly used to form near-homogeneous networks for tissue engineering [28].

Beyond PEG, other synthetic polymers have been explored as crosslinkers. PVA, for example, can act as a crosslinker via its many hydroxyl groups, as in classic PVA hydrogels crosslinked with glutaraldehyde or borate, like Ivalon, mentioned briefly in Section 1 [29]. Similarly, difunctional monomers such as ethylene glycol dimethacrylate (EGDMA) or N,N′-methylenebis(acrylamide) (MBAA) are widely used to crosslink polymeric networks, though these small molecules lack the architectural complexity and flexibility of polymeric crosslinkers. In contrast, dendrimer and star polymer crosslinkers like poly(amidoamine) (PAMAM) dendrimers with multiple surface groups or star-shaped PEGs bring high functionality and can create extremely interconnected networks [30].

In general, synthetic crosslinkers are prized for their predictability, reproducibility, and customizability. One can routinely and facilely adjust their molecular weight, branching, and functional group chemistry to influence the resulting hydrogel’s properties. However, since they often lack intrinsic bioactivity, synthetic crosslinkers are frequently paired with biofunctional peptides or other cues (e.g., RGD sequences for cell adhesion) to impart biological function to the network [53]. The combination of a mechanically robust synthetic crosslinker and a specific biochemical ligand is a common strategy for creating hydrogels that are both appropriately stiff and cell-instructive.

#### 2.1.2. Peptide and Protein Crosslinkers

Peptide- and protein-based crosslinkers utilize their inherent bioactivity to impart cell-interactive or enzyme-responsive properties into the hydrogel network. Short peptides can be engineered with reactive termini (e.g., cysteine thiols, lysine acrylates) to serve as crosslinkers between polymer chains [31]. A common example is the use of protease-degradable peptides as crosslinkers in PEG hydrogels: peptides containing a matrix metalloproteinase (MMP)-cleavable sequence (e.g., GPQGIWGQ) are used to link PEG chains, yielding hydrogel networks that can be degraded by cell-secreted MMPs. It has been shown that MMP-cleavable peptide-crosslinked hydrogels support robust cell invasion, whereas hydrogels with non-degradable crosslinks tend to slow or inhibit cell infiltration, spreading, and migration. This was exemplified by Lutolf et al. [32], who demonstrated that PEG hydrogels crosslinked with MMP-sensitive peptides facilitated significantly deeper fibroblast invasion (~200 µm over 7 days) compared to non-degradable networks (<50 µm). This illustrates how peptide crosslinkers can introduce cell-responsive remodeling behavior, a critical feature for tissue engineering scaffolds that need to accommodate cell infiltration.

Whole proteins or protein domains have also been investigated as crosslinkers. One strategy is to use multivalent proteins with specific binding affinity. For example, the avidin–biotin system has been exploited by decorating polymer chains with biotin and adding avidin or streptavidin (which have four biotin-binding sites) as a tetravalent crosslinker [33]. Another example is using coiled-coil protein motifs that self-assemble: two or more peptides that form a coiled coil can be attached to different polymer backbones, and upon mixing they will dimerize or oligomerize into coiled-coil bundles, effectively crosslinking the polymers [34].

Natural structural proteins themselves can act as the network matrix, but modified forms can also serve as crosslinkers in hybrid systems. Gelatin, a denatured collagen, can be functionalized with reactive groups (e.g., Gelatin-methacrylate, GelMA) [35]. In so doing, the gelatin will contain natural cell adhesion motifs and enzyme-sensitive sites, while allowing for crosslinking via the methacrylate synthetic handle.

#### 2.1.3. Peptoid and Peptidomimetic Crosslinkers

Peptoids (N-substituted glycine oligomers) and other peptidomimetics represent a class of crosslinkers that mimic the structure of peptides but with synthetic backbones or modified linkages that confer unique properties. Peptoids, for example, are similar to peptides but have their side chains attached to the backbone nitrogen, rendering them resistant to proteolytic degradation [36]. As crosslinkers, peptoids combine the sequence programmability of peptides with improved biostability.

Recent work has shown that sequence-defined peptoid crosslinkers can be used to tune hydrogel mechanics with high precision. For instance, a series of helical peptoid oligomers with thiol end-groups was synthesized and used to crosslink hydrogels via thiol–ene chemistry [37]. Because the peptoids were designed to form stable helices, they behaved as semi-rigid rods, such that increasing the peptoid length from 8 to 14 monomers significantly increased the hydrogel’s storage modulus (G′), demonstrating that the peptoid’s molecular stiffness was transmitted to bulk stiffness [38,39]. Moreover, the non-natural backbone endowed these crosslinks with remarkable stability against enzymatic degradation. This allows peptoid-crosslinked hydrogels to maintain integrity in protease-rich environments where a peptide-crosslinked gel would rapidly soften or fail.

Other peptidomimetic systems include β-peptides (peptide analogs with an extra backbone carbon), D-amino acid peptides (chirality-flipped peptides that enzymes do not recognize), and synthetic foldamers that adopt specific shapes. These can be engineered to display specific binding motifs or mechanical responses. For example, a β-sheet-forming analog could be incorporated to create physical crosslinks via intermolecular β-sheet stacking, adding toughness via reversible sacrificial bonds [40].

#### 2.1.4. Polysaccharide-Based Crosslinkers

Polysaccharides, long chains of sugar residues, are another important class of crosslinkers, especially in biomaterials. Many polysaccharides are inherently biocompatible and highly hydrated, making them natural fits for hydrogel fabrication. They can serve either as primary network polymers or as crosslinking agents after chemical modification [3]. Common examples include hyaluronic acid (HA) [41], alginate [42], chitosan [43], and dextran [44]. These molecules often carry multiple functional groups (hydroxyls, amines, carboxylates) along their backbone, which can be used to incorporate crosslinks either ionically or covalently. For instance, alginate, a polysaccharide from seaweed, has blocks of guluronic acid that can chelate divalent cations like Ca^2+^. When CaCl_2_ is added to an alginate solution, Ca^2+^ ions form crosslinks between guluronate residues on different alginate chains, forming ionic junctions that yield a gel [42]. Alginate–calcium gels are widely used for cell encapsulation due to their rapid gelation and mild conditions. However, ionic crosslinks are reversible and can be displaced by chelators or high ionic strength, which means alginate gels often exhibit softening or creep unless further stabilized [45].

Polysaccharides can also be modified for covalent crosslinking. For example, oxidizing alginate generates dialdehyde groups along its chain, which can then form covalent Schiff base bonds (imines/hydrazones) with hydrazide-functionalized polymers. In one study, oxidized alginate was mixed with adipic dihydrazide to form hydrazone crosslinks; by varying the ratio of oxidized alginate to unmodified alginate, researchers could tune the crosslink density and thus the stress relaxation behavior of the hydrogel [46].

A recent comprehensive review by Tang et al. highlights the remarkable potential of polysaccharide-based beads (PSBB) for water purification, underscoring their versatility in adsorbing a wide array of contaminants such as heavy metals, dyes, antibiotics, and oils [47]. The authors systematically explore beads derived from diverse natural polymers including cellulose, chitosan, alginate, starch, lignin, pectin, guar gum, carrageenan, and agar, detailing their preparation methods, physicochemical properties, and adsorption mechanisms. Notably, the review emphasizes the sustainability, biocompatibility, and biodegradability of these materials, positioning PSBB as promising eco-friendly alternatives to conventional synthetic adsorbents. Furthermore, Tang et al. discuss the importance of tuning pore structures, surface functionalities, and crosslinking strategies to enhance adsorption performance, while also identifying key challenges such as mechanical stability, regeneration efficiency, and scalability that must be addressed to advance PSBB toward practical large-scale water treatment applications.

#### 2.1.5. Hybrid and Composite Crosslinker Systems

Hybrid crosslinker systems combine multiple components (often one synthetic and one biological) to create crosslinkers with synergistic properties. The goal is to marry the advantages of synthetic polymers (predictable structure, robust mechanics) with those of bio-inspired elements (specific biochemical responsiveness or hierarchical structure). For example, a PEG chain can be end-functionalized with a biologically active peptide sequence, resulting in a crosslinker that is part synthetic (the PEG provides a flexible spacer and inert backbone) and part biological (the peptide may provide a cleavage site or cell adhesion motif) [48].

Another strategy uses organic–inorganic hybrid crosslinkers. Nano-sized inorganic components (like silicate nanoclays or silica nanoparticles) can function as multifunctional crosslinking nodes when integrated into polymer networks. For instance, silica nanoparticles grafted with polymer chains can bind multiple polymers, effectively acting as highly functional crosslinking sites. A recent study integrated mesoporous silica nanoparticles (MSNs) as covalent crosslinkers into a supramolecular hydrogel network, enhancing its mechanical properties by providing rigid, high-valency junction points [49]. Similarly, exfoliated nanoclays (e.g., Laponite) have been used to physically crosslink PEG via adsorption of polymer segments on the clay surface, yielding extremely tough nanocomposite hydrogels [50].

### 2.2. Structural Typology of Crosslinkers

A crosslinker’s structure (linear, branched, star, side chain, dendritic, etc.) dictates how it connects polymer chains. A linear (telechelic) crosslinker with two reactive ends links exactly two polymer chains. In comparison, multi-arm (branched/star) crosslinkers can attach several chains at once, increasing crosslink density and forming a more robust, interconnected network. Even with the same chemistry, the architecture of a crosslinker can drastically alter hydrogel properties. Here we consider four structural categories: linear crosslinkers, star/branched crosslinkers, side-chain (pendant) crosslinkers, and dendritic crosslinkers. These categories overlap with the chemical designations discussed above (e.g., PEG can be linear or multi-arm; HA allows for side-chain crosslinking), but this section emphasizes how structure specifically impacts network formation and hydrogel mechanics.

#### 2.2.1. Linear Crosslinkers (LX)

Many traditional crosslinkers are linear, with reactive sites at each end of their chain. For example, a PEGDA can link two thiol-bearing polymer chains via Michael addition, or a diamine can crosslink two aldehyde-bearing polymer chains via Schiff base formation. This type of linear crosslinker creates network junctions of functionality 2, meaning each crosslink point in the network joins exactly two strands.

One limitation of using linear (difunctional) crosslinkers is that forming a continuous, percolating network requires relatively long polymer chains at high concentrations [42]. According to classical Flory–Stockmayer gelation theory, crosslinkers with a functionality of 2 demand extremely high extents of reaction to reach the gel point, making network formation more dependent on polymer chain length and density [9,10]. For an equimolar solution we have Equation (1):

(1) $$p_{c}=\frac{1}{f-1}=\;\frac{1}{2-1}=1$$

where $p_{c}$ is the critical extent of reaction at the gel point and *f* is the average functionality of the crosslinkers. Note that this equation implies that all the crosslinkers must react to form a gel, whereas for a tetrafunctional solution, only a third of the reactive groups would need to react to reach $p_{c}$.

The resulting networks often have relatively large mesh sizes and may be softer if the base polymers are flexible. For example, hydrogels crosslinked by long linear PEGs can be quite compliant because each PEG crosslinker acts like a pliable spacer between junctions [51]. If shorter linear crosslinkers or small-molecule crosslinkers (e.g., MBAA) are used, the network can be tighter, but often these small crosslinkers introduce local network irregularities (e.g., very short loops or dangling ends that do not contribute to network connectivity).

Linear crosslinkers do have advantages: they are often easier to synthesize and characterize (having only two reactive ends) and can yield very homogeneous networks if the polymerization is well-controlled. A notable case is the Tetra-PEG hydrogel system, which effectively uses linear linkers in an end-linking strategy: two four-arm PEGs (each end-terminated with mutually reactive groups) end-linked to each other, and the connecting bridges between four-arm nodes are essentially linear PEG chains. This system achieves an extremely homogeneous network structure that approaches the ideal Flory–Stockmayer network with highly reproducible mechanical properties [20].

#### 2.2.2. Star (SX) and Branched Crosslinkers

Star and branched crosslinkers have multiple reactive functionalities (>2) emanating from a central core or along a branched backbone. Common examples are multi-arm PEGs (e.g., three-arm, four-arm, eight-arm PEG), branched multi-functional polymers, or proteins like streptavidin. The defining feature is that a single molecule can connect three or more polymer chains, acting as a junction with functionality equal to its number of arms/reactive sites.

Using star/branched crosslinkers generally increases the crosslinking density and network connectivity at a given polymer concentration. This typically leads to stiffer, more elastic hydrogels. For instance, switching from a linear bifunctional PEG to a four-arm PEG crosslinker in a given hydrogel raises the shear modulus, and going to an eight-arm PEG can boost it further. In the context of dynamic hydrogels, one study on hydrazone-crosslinked alginate found that hydrogels with star crosslinkers (four-arm) were significantly stiffer and exhibited slower stress relaxation than those crosslinked with linear crosslinkers, due to the higher effective crosslink valency [27]. Moreover, the mechanical properties of the star-crosslinked hydrogels were more robust to perturbations like competing side reactions, whereas linear-crosslinked hydrogels were more sensitive (losing more stiffness if some crosslinks were consumed by side reactions).

Branched crosslinkers also affect network topology by introducing branching points that can distribute stress. They reduce the relative number of chain ends in the network (since each crosslinker ties up several chain ends into one node), which can decrease the fraction of dangling or loose ends and thereby enhance the plateau modulus. An idealized case is the tetra-PEG end-linked network mentioned for linear crosslinkers: it can also be viewed as a network of four-functional junctions (each four-arm PEG is a junction point). Once again, such tetra-PEG gels achieve highly uniform and tough networks [20].

#### 2.2.3. Side-Chain Crosslinking (SCX)

Side-chain crosslinking (SCX) refers to the scenario where crosslinkable groups are distributed along the polymer backbone as pendant functionalities, rather than only at the chain ends. In this architecture, each polymer chain can form many linkages at various points along its length. Side-chain crosslinking effectively turns the polymer into a multi-valent crosslinker itself. For example, consider an HA chain where some fraction of the disaccharide units is modified with methacrylate groups (HAMA). During photopolymerization, those methacrylate side groups on one HA chain can crosslink with methacrylate groups on neighboring HA chains, forming a network. In this case, each HA chain is crosslinked at numerous points along its length. Similarly, GelMA (gelatin methacrylate) has dozens of methacrylate groups per protein chain, leading to a highly crosslinked protein network upon UV exposure. As a result, SCX networks can be stiffer and less elastic (more brittle) because the polymer strands between crosslinks are shorter and more constrained. Recent work by Lin and colleagues systematically compared SCX versus linear crosslinking in alginate hydrogels [27]. They attached aldehyde groups either along the alginate backbone (many per chain) or only at the chain ends and crosslinked each with hydrazide linkers. Hydrogels with SCX exhibited higher initial stiffness and significantly slower stress relaxation than those with only end linkages. Essentially, tethering each polymer at multiple points restricted chain mobility and made the network more solid-like (stress relaxation was slower because chains could not easily redistribute stress).

It is important to note that SCX can lead to intra-chain loop formation if two reactive groups on the same polymer react with each other or via a short crosslinker. Such loops do not contribute to the network connectivity and can be viewed as defects that reduce the effective crosslink density. The probability of loop formation increases with very high functional group density. To mitigate this, one can use strategies like dilute functional group distribution or use of bulky crosslinkers that prefer inter-chain linking.

Many natural polymer networks resemble SCX architecture. For instance, fibrillar collagen, aside from end crosslinks, also forms lateral crosslinks through enzymatic action and physical entanglements along the fiber length, creating a kind of side-chain or inter-fibril crosslinking that contributes to tissue stiffness [24]. Utilizing SCX architecture may be considered for synthetic hydrogels aiming to mimic the high crosslink density of native ECM.

#### 2.2.4. Dendritic Crosslinkers

Dendritic crosslinkers represent a highly branched architectural class characterized by a tree-like structure with multiple generations emanating from a central core. These macromolecules possess a high density of terminal functional groups, often ranging from tens to hundreds depending on the generation and backbone chemistry. This exceptionally high functionality enables a single dendritic crosslinker to engage in extensive multivalent interactions, thereby dramatically increasing the potential crosslinking density of a hydrogel network.

Among the most widely studied dendritic crosslinkers are PAMAM dendrimers. For instance, a generation 4 PAMAM dendrimer presents 64 terminal amines, allowing it to simultaneously bind numerous polymer chains via Michael-type additions, Schiff base reactions, or other orthogonal chemistries. When mixed with complementary functionalized polymers (e.g., acrylate-terminated PEGs or thiolated polysaccharides), such dendrimers form densely crosslinked and mechanically robust hydrogel networks. Due to the localized concentration of reactive groups, gelation can proceed rapidly, and the resulting networks may exhibit extremely low swelling ratios and high elastic moduli, making dendritic systems particularly useful in load-bearing or shape-retentive biomaterials.

However, the utility of dendritic crosslinkers comes with several design considerations. The uniformity of network formation is highly sensitive to the spatial distribution and accessibility of terminal groups on the dendrimer surface. Incomplete or sterically hindered reactions can lead to inhomogeneous crosslinking, resulting in network defects such as unreacted chain ends, dangling ends, or unincorporated dendrimers. Moreover, the presence of high local functionality can exacerbate the formation of intramolecular loops or microgels, reducing the effective connectivity and undermining mechanical integrity. To mitigate these issues, careful control over stoichiometry, crosslinker concentration, and reaction kinetics is essential.

## 3. Crosslinking Chemistry, Kinetics, and Dynamics

The chemistry, kinetics, and dynamics of crosslinking reactions collectively govern how a hydrogel forms, evolves, and responds under various conditions. Crosslinking chemistry defines the type of bonds formed (covalent, ionic, etc.) and determines key properties such as stability, degradability, and biocompatibility of the resulting hydrogel. Kinetics dictate how quickly the network forms, which is especially critical for applications involving in situ gelation or cell encapsulation, where rapid yet controlled gelation is essential to prevent cell sedimentation or toxicity. Meanwhile, the dynamic behavior of crosslinks (the timescale on which the bonds form and break) shapes the hydrogel’s ability to self-heal, adapt, or remodel in response to environmental cues. Together, these factors create a rich design space for tailoring hydrogel performance, offering precise control over both the structure and function of the material over a variety of timescales. See Table 2 for a complete list of the crosslinking mechanisms and chemistries discussed in this section.

### 3.1. Covalent Crosslinking Mechanisms

Covalent crosslinking involves the formation of usually permanent chemical bonds between polymer chains. These bonds lock in the network connectivity once formed, yielding hydrogels that behave as elastic solids (at least on timescales shorter than any bond breakage or polymer degradation). Covalent crosslinking can be initiated through various chemical routes. Key mechanisms include photopolymerization [46] (often via free-radical reactions), click chemistry conjugations [79] (rapid coupling reactions with high specificity), thiol–ene reactions [54] (a step-growth photopolymerization), Michael-type additions [80], and enzyme-mediated additions [81]. The kinetics of these crosslinking reactions strongly influence the microstructure and properties of the resulting network.

#### 3.1.1. Photopolymerization and Click Chemistry Approaches

Photopolymerization is a fundamental technique for forming covalently crosslinked hydrogels, typically employing UV-A (320–400 nm) or visible light (400–500 nm) wavelengths to activate photoinitiators. The choice of wavelength is crucial for cytocompatibility, as longer wavelengths in the visible range reduce DNA damage and cell stress, enabling encapsulation of sensitive cell types. Photopolymerization is of particular interest when spatial and temporal control is desired. In typical photopolymerization, polymers or multi-functional monomers are mixed with a photoinitiator and are exposed to light. The initiator generates radicals that propagate polymerization and crosslinking, converting a liquid precursor into a crosslinked gel. PEGDA and GelMA are classic macromers that form hydrogels via photoradical crosslinking and are widely used in 3D cell culture and tissue engineering.

The appeal of photopolymerization lies in its speed and precision: gelation can occur within seconds to minutes upon illumination, and by using patterned light one can create spatial variations in crosslink density (enabling micropatterned scaffolds) [82]. For instance, a liquid precursor can be injected into a tissue defect and then cured in place with light to form a hydrogel implant with a shape defined by a photomask. Photopolymerization also allows in situ gelation in a minimally invasive manner.

One downside of traditional chain-growth photopolymerizations is that they can lead to network heterogeneities [55]. Polymerization can outrun molecular diffusion, causing locally high crosslink densities (microgels) that subsequently connect into an inhomogeneous network. Additionally, oxygen can inhibit radical polymerization by quenching free radicals, leading to incomplete network formation and leaving behind unreacted functional groups [56]. These unreacted components contribute to the sol fraction, or the portion of material that remains soluble and is not incorporated into the crosslinked network. These effects often manifest as spatial variation in mechanical properties and lower effective crosslink conversion. In contrast, step-growth photopolymerizations tend to proceed more uniformly. Indeed, studies have found that step-growth networks formed by photochemistry are more homogeneous and reach higher functional group conversion than analogous chain-growth acrylate networks [54].

Click chemistry refers to a set of efficient, modular reactions that proceed under mild conditions with high yield. Many click reactions have been adapted for hydrogel crosslinking, either with or without photoinitiation. A prime example is the strain-promoted azide-alkyne cycloaddition (SPAAC), a copper-free click reaction between an azide and a cyclooctyne. This reaction is bio-orthogonal and releases no toxic byproducts, making it ideal for cell-encapsulation. For instance, an injectable PEG hydrogel fabricated via SPAAC chemistry was formed by rapidly mixing multi-azide PEG and multi-cyclooctyne PEG, resulting in gelation within minutes without a catalyst [57]. The resulting network was biocompatible and degradable, and it formed in situ in mice with minimal inflammatory response, exemplifying how click chemistry enables rapid, catalyst-free crosslinking in physiological settings.

#### 3.1.2. Thiol–Ene Reaction Systems

A quintessential click reaction in hydrogels is thiol–yne or thiol–ene coupling. Often photoinitiated, thiol–ene reactions combine the advantages of photopolymerization and click chemistry: they are orthogonal, proceed quickly under light, and result in step-growth network formation which yields a homogeneous structure. In a thiol–ene crosslinking system, a thiol group (-SH) reacts with carbon–carbon double bond, typically under UV light and with a photoinitiator. The mechanism proceeds via a radical-mediated addition: a photogenerated thiyl radical adds to the carbon–carbon double bond, forming a carbon-centered radical, which then abstracts a hydrogen from another thiol, regenerating a thiyl radical and propagating the reaction.

Thiol–ene crosslinking tends to distribute crosslinks more evenly through the material. All functional groups react more or less concurrently throughout the gelation process (especially if stoichiometrically balanced), resulting in networks with homogeneous mesh sizes and high functional group conversion. Many studies have found thiol–ene gels to have superior network regularity compared to acrylate-based gels, for example [54]. Additionally, thiol–ene reactions can be carried out with low-toxicity photoinitiators (e.g., lithium acylphosphinate, LAP) under long-wavelength UV or visible light, minimizing cell damage. Moreover, thiol–ene reactions are not inhibited by oxygen to the same extent as acrylate radical polymerizations, which means they can polymerize efficiently in tissue culture conditions without deoxygenation.

Thiol–ene crosslinking has been exploited in many applications requiring fast gelation and biocompatibility: for instance, as bioinks in 3D printing [83] (where fast, consistent crosslinking is needed for shape fidelity), or in forming cell-laden hydrogels in microfluidic devices [84] (where cytocompatibility and rapid set times are crucial). Additionally, the thiol–ene reaction’s tolerance for secondary functionalization allows for sequential reactions, e.g., first form a thiol–ene hydrogel, then introduce another set of crosslinks or modifications (like attaching a fluorescent tag or RGD via residual thiols).

#### 3.1.3. Michael Addition and Other Addition Reactions

Michael addition in hydrogels usually involves a nucleophilic thiolate or amine adding to an electron-deficient vinyl (such as acrylate, methacrylate, or vinyl sulfone). This reaction proceeds readily under physiological conditions without external initiators, making it popular for in situ crosslinking of hydrogels in the presence of cells. A classic example is PEG–vinyl sulfone (PEG-VS) crosslinked by a multi-thiol peptide via a base-catalyzed Michael addition: the thiol from a cysteine on the peptide reacts with the double bond of the vinyl sulfone to form a thioether linkage. Gelation occurs as PEG-VS molecules connect with the multi-thiol (often a tri- or tetra-thiol peptide), forming a network. This reaction can be tuned to gel in times ranging from seconds to hours by adjusting pH/catalyst (often triethanolamine), providing flexibility for different applications.

Michael-type crosslinking is highly cytocompatible since it does not generate radicals or require UV light. Additionally, bioactive cues can be easily integrated: one can mix a thiol-bearing RGD peptide into a PEG-VS hydrogel formulation, and the RGD will tether into the network during crosslinking. An interesting consideration in these systems is competition between crosslinking and functional group conjugation, e.g., if RGD (monothiol) competes with dithiol crosslinker for PEG-VS, it can reduce crosslink density, so the relative stoichiometries must be considered in the hydrogel formulation.

Beyond thiol-based Michael additions, amine-based Michael additions (also known as aza-Michael additions) are commonly used for hydrogel production. For instance, primary amines (from lysine residues or diamines) can add to acrylates, forming β-amino esters. This approach has been used to form poly(β-amino ester) hydrogels that degrade hydrolytically [58]. Enzymatic methods can also create addition-type crosslinks: transglutaminase catalyzes bonds between glutamine and lysine side groups (as in fibrin crosslinking) [59], and horseradish peroxidase with hydrogen peroxide (H_2_O_2_) creates dityrosine linkages between tyrosine-functionalized polymers [60]. These enzymatic crosslinking methods effectively act as addition reactions.

Hydrogels formed by Michael addition often have distinct properties: they lack chain polymerization byproducts (no long kinetic chains or heterogeneous microgels), often resulting in lower shrinkage and uniform networks. However, network defects can arise if the reaction is too slow (allowing cyclization) or if the stoichiometry is imbalanced. Typically, one designs the gel to form at a controllable rate (on the scale of minutes), which is fast enough to trap a homogeneous mixture but slow enough to mix and cast before gelation.

A noteworthy benefit is that Michael addition permits injection and subsequent rapid gelation. Some Michael systems are injectable as two-component mixtures that gel shortly after mixing. For example, thiolated HA and PEGDA can be co-injected in the liquid state, allowing the gel to set in situ [61].

#### 3.1.4. Effect of Crosslinking Kinetics on Resulting Network Structure and Hydrogel Properties

The rate of crosslink formation can profoundly impact hydrogel network structure and properties. When crosslinking is very rapid, the polymer chains become fixed in position quickly, potentially locking in spatial heterogeneities present at the moment of gelation. For example, extremely fast photopolymerizations can produce concentrated regions of crosslinks (where local polymer density was initially higher) before the network has time to equilibrate, leading to non-uniform mesh size distribution. Conversely, slower crosslinking allows polymer chains more time to diffuse and rearrange as the network forms, often yielding a more homogeneous crosslink distribution.

Crosslinking kinetics also influence the time of gelation (the time to reach the gel point). A very fast-crosslinking system reaches the gel point at low conversion of functional groups, meaning many unreacted groups remain trapped in the gel state (some will later react, some may remain as dangling ends). A slower gelling system might not solidify until higher conversion, potentially resulting in a more fully crosslinked network. If the goal is to maximize crosslink conversion (to minimize leachable monomers, for instance), a slower reaction or post-cure steps may be preferred.

From a mechanical perspective, faster gelation can lock in higher internal stresses and can yield a stiffer initial material. Slower gelation often produces a material that is slightly softer (if some reactive groups are lost to loops) but can be tougher due to fewer stress concentrators. For example, extremely rapid photogellation can sometimes lead to brittle hydrogels, whereas a slightly slower curing allows for a tougher, more defect-free network.

In practice, it is critical to tune crosslinking kinetics to the specific application by adjusting initiator concentration, temperature, catalyst, or using retarders. In situ-forming hydrogels need a balance: gelation should be fast enough to stay in place (not wash away from the injection site), but not so fast that it cannot be delivered or shaped. For instance, a clinical-grade fibrin glue sets in seconds (driven by thrombin concentration), whereas a cell-encapsulating PEG-peptide hydrogel might be formulated to gel in a few minutes which is fast enough to maintain a uniform cell distribution but not so slow as to allow significant sedimentation before network stabilization.

As a practical demonstration, Lou and coworkers devised a strategy to decouple crosslinking kinetics from crosslink density using a catalyst in a hydrazone-crosslinked hydrogel [62]. By adding a small organic catalyst, they accelerated the dynamic covalent exchange (see Section 3.2) without changing the number of crosslinks at equilibrium. This controls the rate of gelation without changing the resulting modulus.

### 3.2. Dynamic Covalent Crosslinking

**Figure 2:**
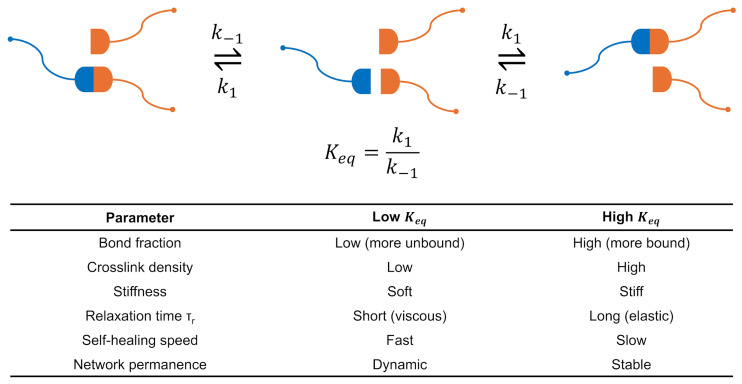
Reversible bond exchange governing dynamic equilibrium in hydrogel networks.

Dynamic covalent crosslinks are covalent bonds that can break and re-form reversibly under certain conditions, endowing hydrogels with malleable, time-dependent properties (see Figure 2). Hydrogels utilizing such bonds behave like elastic solids on short timescales but can relax stress and flow over longer times as bonds exchange. These are sometimes called covalent adaptable networks (CANs). Dynamic covalent chemistry in hydrogels offers a means to mimic the viscoelastic and self-healing nature of living tissues within a chemically crosslinked material.

#### 3.2.1. Principles of Reversible Covalent Bonds in Hydrogels

A reversible covalent bond has the ability to dissociate and re-associate, either spontaneously or in response to a stimulus. In a hydrogel network, if crosslinks are reversible, the network connectivity is not static: bonds can break and later re-form, possibly in a different configuration. This imparts viscoelastic behavior and often self-healing ability, due to the network’s ability to rearrange under stress. Several types of reversible covalent bonds have been employed for dynamic crosslinking:

Schiff base bonds [63] (imines, hydrazones, oximes): These form by condensation of an aldehyde with a nucleophile like an amine, hydrazide, or aminooxy. Imine bonds (C=N) are reversible via hydrolysis. Hydrazone bonds (from aldehyde + hydrazide) and oxime bonds (aldehyde + aminooxy) are more stable than simple imines but still dynamic, especially under slightly acidic or basic conditions.

Disulfide bonds [65] (-S-S-): Formed by oxidation of thiols, they can be reduced back to thiols. In the absence of reducing agents, disulfides can also undergo thiol–disulfide exchange with free thiols. In hydrogels, this exchange allows for network rearrangement.

Boronate ester bonds [66]: Formed between diols and boronic acids, these bonds dynamically break and re-form via transesterification with other diols or water. Their stability is tunable by pH (they are stronger at slightly alkaline pH where boronate is ionized). They can respond to stimuli like glucose (which competes for boronic acid binding) or pH shifts.

Dynamic Diels–Alder adducts [67]: Certain Diels–Alder linkages (like furan–maleimide) are reversible at elevated temperature. Hydrogels have been formulated wherein Diels–Alder adducts dissociate by heating (allowing the gel to flow or heal) and re-form by cooling.

Enamine bonds or reversible covalent enolates [85]: Emerging chemistries like reversible covalent crosslinks through enamine formation or alkoxyamine (as in vitrimers) are also possible in hydrogels, though less common in aqueous systems.

The unifying principle is that these bonds are stable under normal conditions (so the hydrogel is solid at rest) but can reconfigure via bond exchange. The kinetics of bond breakage (k_off_) and re-formation (k_on_) determine how quickly the network responds to stress. If the exchange is fast relative to the experimental timescale, the material behaves more liquid-like; if the exchange is slow, it will be more solid-like.

Dynamic covalent hydrogels often exhibit stress relaxation: if you apply a strain and hold it, the stress decays as crosslinks break and re-form, redistributing the load. They also often show self-healing: if cut in two and the surfaces are brought back together, the crosslinks across the interface can re-form, healing the hydrogel.

A relevant example is a hydrazone-crosslinked HA hydrogel: it forms quickly as an aldehyde-modified HA reacts with adipic dihydrazide. Over time, the hydrazone bonds can exchange (especially if slightly acidic), allowing the network to relax any built-up stress and even permit cells to remodel the matrix by pushing through (the bonds break and re-form around the cell).

The equilibrium of dynamic bonds is also a key design parameter in these reversible systems. If the bond is very stable (k_on_ >> k_off_), most crosslinks will be intact at any given time, and the hydrogel behaves more like a solid with slight viscoelasticity. If the bond equilibrium favors the unbonded state (k_on_ << k_off_), the hydrogel may behave very soft or even fluid-like.

#### 3.2.2. Stress Relaxation Behavior in Dynamic Covalent Crosslinked Hydrogels

Stress relaxation behavior is a key metric for viscoelastic materials and is often quantified by a relaxation time (τ) or a spectrum of relaxation times. From a biological perspective, stress relaxation in hydrogels is critical for cell mechanotransduction, as will be discussed in detail in later sections. Cells in a purely elastic hydrogel (no relaxation) experience a sustained resistance when they spread or proliferate, which can inhibit certain functions. In contrast, in a viscoelastic gel, the initial resistance can decay, and the cell can gradually push the network and create space. Chaudhuri et al. showed that stem cells spread and differentiate more in hydrogels with faster stress relaxation, even without a change in bulk stiffness [86]. In their experiments, mesenchymal stem cells (MSCs) in a fast-relaxing hydrogel (τ~1 min) spread and exhibited stress–fiber formation more similarly to in natural tissues, whereas in an elastic control (no relaxation) they remained round. This underscores that it is not just stiffness but also stress relaxation behavior that influences cell fate.

Another manifestation of hydrogel viscoelasticity is creep: under a constant stress, the material will continue to deform over time as bonds rearrange [87]. Rheologically, dynamic covalent hydrogels typically show a G′ that decreases with decreasing frequency and a loss modulus (G″) that can be significant at low frequencies, indicating fluid-like behavior on long timescales. The point at which G″ surpasses G′ (the crossover point) corresponds to a timescale where the hydrogel transitions from a primarily solid-like to a primarily liquid-like response, which can be related to the dominant relaxation time of the network.

From a materials design perspective, one can tailor the stress relaxation profile by mixing dynamic crosslinkers of different kinetics. A hydrogel with two distinct dynamic crosslink chemistries (say, a fast boronate and a slower hydrazone in the same network) will have a bimodal relaxation spectrum, relaxing a certain amount quickly (via boronates) and the rest more slowly (via hydrazones). This can be leveraged to fine-tune how a material responds over multiple timescales—a concept known as hierarchical viscoelasticity, which is relevant to mimic biological tissues that have collagen (slow relaxing) and proteoglycan (fast relaxing) components.

#### 3.2.3. Relationship Between Bond Exchange Kinetics and Viscoelasticity

The viscoelastic properties of dynamic covalent hydrogels are inherently tied to the kinetics of bond exchange. In general, the characteristic relaxation time of a network is inversely related to the rate at which crosslinks can rearrange (k):

τ ≈ 1/k

If bonds break and re-form rapidly, the network can relax stress quickly (small τ); if bonds exchange slowly, the network retains stress longer (large τ). This concept is often described using a Maxwell model for a simple viscoelastic system: an elastic spring in series with a dashpot. The dashpot’s viscosity is related to bond kinetics—faster kinetics equate to lower viscosity. This has been evaluated in depth in recent years by Anseth and colleagues [88].

Mihajlovic et al. demonstrated control over viscoelasticity by designing a hydrogel with two types of crosslinks: permanent covalent Diels–Alder links and dynamic hydrazone links [64]. By changing the ratio of dynamic to permanent crosslinks, they could dial in the viscoelastic response: with more dynamic bonds, the hydrogel had more pronounced stress relaxation, whereas with more permanent bonds, the gel retained more stress. Significantly, the immediate elastic modulus was governed mostly by the total crosslink density (sum of permanent + dynamic), while the relaxation ratio (fraction of stress relaxed) was governed by the proportion of dynamic bonds.

For thermally reversible bonds like certain Diels–Alder or disulfide exchanges, raising temperature accelerates bond exchange, thus making the hydrogel more fluid-like. This can be used to create, for example, a hydrogel that is firm at room temperature but becomes viscoelastic or even liquid at slightly elevated temperature and then re-forms when cooled. Visible or UV light exposure can similarly modulate kinetics for photo-responsive dynamic bonds (like diselenide which exchanges under light [89]).

### 3.3. Non-Covalent Crosslinking Interactions

Non-covalent crosslinks are physical interactions that hold polymer chains together without forming permanent bonds. These include hydrogen bonds, ionic interactions, crystallite formation, π-π stacking, host–guest inclusion complexes, hydrophobic associations, and metal–ligand coordination, among others. Hydrogels built on these interactions are often called “physical” or “supramolecular” hydrogels. They tend to be inherently dynamic, as these bonds can break and re-form under physiological conditions. Non-covalent crosslinking offers reversibility, self-healing, and stimuli-responsiveness, and these interactions can be used alone or combined with covalent crosslinks to create hybrid networks with complex mechanical behavior.

#### 3.3.1. Hydrogen Bonding Networks as Crosslinking Motifs

Hydrogen bonds are ubiquitous in polymers and can serve as transient crosslinks in hydrogels. While single hydrogen bonds are weak, multiple H-bonds acting cooperatively can produce substantial crosslinking strength. For example, PVA chains exhibit significant hydrogen bonding, especially upon freezing/thawing [68]. These PVA cryogels are physically crosslinked by crystalline regions stabilized by hydrogen bonds and have considerable strength and elasticity.

Another example is self-complementary motifs like ureido-pyrimidinone (UPy), which form quadruple H-bonded dimers [69]. When UPy groups are grafted onto a polymer, they can dimerize and effectively crosslink two chains. UPy-based hydrogels exploit this with multiple UPy stickers per chain, leading to highly elastic yet self-healing networks.

Hydrogels rich in hydroxyl and amide groups often owe much of their structure to hydrogen bonding. Gelatin forms a physical gel as it cools due to partial renaturation of collagen triple helices (stabilized by H-bonds). Similarly, cellulose nanofibrils can H-bond with each other to create fibrillar networks in hydrogels. These H-bond-driven interactions are usually reversible with temperature or solvent composition.

Hydrogen-bonded networks tend to show significant viscoelasticity. The bonds break and re-form on a broad range of timescales, giving rise to time-dependent stress relaxation. They can also be quite tough: some double-network hydrogels include an H-bond-rich network as the sacrificial soft network that yields and dissipates energy. For example, poly(2-acrylamido-2-methylpropanesulfonic acid) (PAMPS) is a polymer that forms H-bonded clusters that serve as sacrificial bonds, making the material tough and strain–stiffening [70].

#### 3.3.2. Host–Guest Interactions and Their Influence on Network Dynamics

Host–guest chemistry involves the non-covalent inclusion complexation between two molecules: a “host” macrocycle and a “guest” molecule that fits into the host’s cavity. Common host–guest pairs in hydrogels are cyclodextrin (host) with hydrophobic guests (e.g., adamantane), or cucurbiturils with various guests. These complexes can have very high binding affinities (dissociation constants in the µM to nM range), effectively acting as transient crosslinks that can dissociate and reassociate.

Cyclodextrin–adamantane (CD-Ad) crosslinking is a widely used motif. β-cyclodextrin is a toroidal sugar macrocycle that can host hydrophobic guests. If one polymer is decorated with CD and another with Ad, mixing them will form a hydrogel as CD-Ad inclusion complexes crosslink the two polymers [71]. These host–guest hydrogels are shear-thinning and self-healing: under shear, some CD-Ad complexes disengage (allowing flow), and upon resting, the guests find hosts again and the hydrogel re-forms.

Another interesting host–guest system is cucurbituril (CB) complexes. Cucurbit(8)uril (CB(8)), for instance, can host two guest molecules simultaneously, often forming a stable ternary complex. Researchers have exploited CB(8) to crosslink two polymers each bearing different guest moieties (for example, viologen, and naphthalene). CB(8) can bind both, effectively locking the two polymer chains together. These hydrogels tend to relax stress more slowly due to their high stability but still allow self-healing over longer times [72].

#### 3.3.3. Metal–Ligand Coordination in Crosslinked Networks

Metal–ligand coordination bonds are another class of physical crosslinks that can impart dynamic and often high-strength interactions. Many polymers can be functionalized with ligands (e.g., histidine, terpyridine, catechol) that chelate metal ions and form coordinate crosslinks. These bonds can be quite strong but are reversible: the metal can dissociate or exchange ligands under certain conditions (changing pH, redox state, or by competitive binding).

A well-known example is alginate crosslinked by divalent cations like Ca^2+^ (already discussed in Section 2.1.4). Calcium–guluronate coordination is essentially ionic in nature. Another common example is histidine–Ni^2+^ coordination: polymers (or peptides) with multiple histidines can bind Ni^2+^ or Zn^2+^ strongly, forming network junctions [73]. These are used in some self-healing materials where imidazole-metal bonds break and re-form.

Another extensively studied system involves catechol and Fe^3+^. Catechol can bind Fe^3+^ in mono-, bis-, or tris-coordinated complexes. At higher pH (~8), it preferentially forms bis- and tris-catechol complexes. The tris-catechol-Fe^3+^ complex is weaker (each catechol–Fe bond is shorter-lived under force) than the bis-catechol [74]. Lee et al. used the following in hydrogels: under acidic conditions, each Fe^3+^ crosslinks mostly two catechols (forming a bridging complex that is very strong, yielding a stiff gel), whereas at higher pH each Fe^3+^ might bind three catechols (each bond a bit weaker, overall giving more viscoelastic and dissipative behavior) [75,76]. These Fe–catechol networks are also self-healing: when a crack forms, the Fe^3+^ can re-coordinate with nearby catechols once the stress is removed, allowing repair over time.

Metal–ligand crosslinks are often sensitive to external stimuli: adding a chelating agent like ethylenediaminetetraacetic acid (EDTA) will sequester the metal and cause the hydrogel to dissociate, which offers utility in drug delivery applications. Redox changes can switch metals between states (for example transforming Fe^2+^ to Fe^3+^) that have different coordination preferences, thus altering crosslinking. Even simply changing the metal can change bond strength (e.g., Ca^2+^ vs. Ba^2+^ in alginate, or Ni^2+^ vs. Cu^2+^ with histidine ligands).

Mechanically, coordination bonds often act as sacrificial bonds that enhance toughness. For instance, a coordination-crosslinked hydrogel can exhibit a high initial modulus, but upon straining, these bonds break, allowing the material to extend significantly. The broken bonds then re-form, enabling self-healing and internal stress relaxation (much like dynamic covalent bonds).

#### 3.3.4. Combining Covalent and Non-Covalent Interactions for Complex Mechanical Responses

More advanced hydrogel systems have considered combining multiple crosslinking mechanisms to achieve tailored mechanical profiles. By layering these interactions, one can create materials that, for example, have a stable permanent network to maintain long-term shape, plus a reversible network to dissipate stress, plus perhaps another transient network to respond to a specific stimulus. Such multi-network or interpenetrating designs can produce exceptional mechanical performances and tunabilities toward targeted applications.

The premiere example is the double-network (DN) hydrogel concept, which pairs a brittle, tightly crosslinked network with a soft, loosely crosslinked network. The first network fractures in a controlled way under stress, while the second network holds the material together. This combination yields extremely high toughness (orders of magnitude higher than either network alone). Although classic DN gels use two covalent networks, recent variants use a physical second network (e.g., hydrogen-bonded or ionically crosslinked) to allow self-healing. For instance, hydrogen-bonded ionic clusters have been used as the second network in a DN gel to give it self-healing properties while retaining high toughness [77].

Another approach is the dual crosslinking of the same polymer with both covalent and non-covalent bonds. For example, one can prepare a hydrogel with polymer chains that are chemically crosslinked by a small percentage of covalent bonds to ensure the gel does not dissociate, and in addition, have many reversible ionic or host–guest crosslinks to provide viscoelasticity. These dual-crosslinked networks often show a combination of high elastic modulus (from the covalent backbone) and high toughness or extensibility (from the breaking of reversible bonds). Sun et al. demonstrated this concept with an alginate/polyacrylamide dual network: alginate (ionically crosslinked by Ca^2+^) provides viscoelastic, sacrificial bonds, while polyacrylamide (covalently crosslinked) provides elasticity; the result was a hydrogel that could stretch 20× its length while also resisting breakage far better than either constituent alone. The ionic alginate could relax stress (preventing crack propagation) and then re-zip (giving partial self-healing and internal energy dissipation), whereas the polyacrylamide prevented complete flow and maintained shape memory [78].

Mechanical experiments on these combined systems often show multi-phase relaxation: a rapid initial drop in stress (attributed to fast reversible bonds like H-bonds or host–guest breaking), followed by a slower decay (from dynamic covalent bonds, for example), and a residual stress plateau. By adjusting the composition, one can tune the proportions of these phases. For example, by increasing the fraction of permanent crosslinks, the final plateau increases. By using stronger or more numerous reversible crosslinks, one can increase the time or extent of the slow relaxation phase.

Finally, combining covalent and non-covalent interactions can yield extreme properties like shape-memory or stimuli-switchable mechanics. A shape-memory hydrogel can be made with a permanent network to fix a temporary shape and a thermal-responsive reversible network to allow deformation and locking/unlocking of the shape. One such design used crystalline domains (melting at a trigger temperature) alongside a covalent network: the material could be deformed above the melting temperature (physical crosslinks disengaged), fixed into a new shape by cooling (re-forming crystals), and then when reheated, it returned to its original shape due to the covalent network’s memory. Similar principles can be applied with host–guest networks that lock at one condition and unlock at another [51].

## 4. Structure–Property Relationships

The mechanical and viscoelastic properties of hydrogels are heavily influenced by modifications in their structure and network conformation. Varying certain crosslinker design elements, like those discussed above, result in the ability to widely tune the resulting mechanical and viscoelastic properties of the hydrogel formulation. However, to fully realize this utility, one must imagine how these variables can be tuned to optimize hydrogels for a given application. In particular, this section will focus on the design of hydrogels for biomedical applications.

### 4.1. Network Connectivity Effects

The connectivity of a hydrogel network is dictated by the type of crosslinking, length of crosslinkers, and degree of crosslinking as discussed in Section 1, Section 2 and Section 3. Notably, these properties are often the most critical in biomedical applications (i.e., 3D cell culture, tissue engineering, and drug delivery). This section explores how one can use crosslinking parameters to determine mesh size, mechanics, and eventually biomedical outcomes for a given hydrogel system.

#### 4.1.1. Crosslink Density Directly Impacts Hydrogel Mechanics

Crosslinking density refers to the quantity of crosslinks connecting polymer chains within a given unit volume in a polymer network [90]. Physicochemical and functional properties of hydrogels, like biodegradability, swelling, and mechanical properties, can be modulated by tuning crosslink density [91,92]. These factors are critical in use cases across the biomedical field and facilitate the development of platforms for drug delivery, transdermal therapies, cell encapsulation, regenerative medicine, tissue engineering, and in situ forming implants, as well as long-term bioadhesive dressings [93,94].

**Figure 3:**
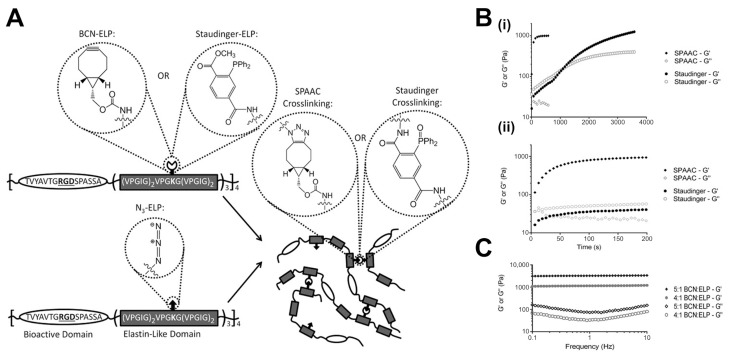
Tunable mechanical properties in recombinant ELP hydrogels through modular crosslinking design. (**A**) Schematic representation of strain-promoted azide-alkyne cycloaddition (SPAAC) used to crosslink azide-functionalized elastin-like proteins (ELPs) with BCN-bearing counterparts. (**B**) Rheological time sweep curves comparing gelation kinetics of SPAAC- and Staudin ger-crosslinked ELPs at two time scales ((**i**) and (**ii**), respectively). (**C**) Frequency sweep data showing the effect of crosslinking stoichiometry (BCN:ELP ratio). Reproduced with permission from Madl et al., Advanced Functional Materials; published by John Wiley & Sons, 2016 [96].

One of the most fundamental biomedical applications of hydrogels is tissue engineering, due to its important role in the manufacturing of functional three-dimensional matrices that function as biological substitutes or enhancers for damaged tissues or organs [95]. In a representative study [96], a synthetic recombinant elastin-like protein (ELP) hydrogel was developed and crosslinked through SPAAC. This bio-orthogonal reaction occurred between azide-functionalized ELPs and bicyclononyne (BCN)-modified counterparts, allowing efficient and catalyst-free formation of covalent crosslinks under mild conditions as depicted in Figure 3A. This system facilitated the precise tuning of crosslink density by varying the stoichiometry of the reactive groups. Gelation kinetics were monitored using oscillatory rheology in real time. As shown in Figure 3B, a sharp increase in G′ immediately after mixing indicated rapid gelation kinetics. Figure 3C demonstrated that increasing the BCN:ELP ratio from 4:1 to 5:1 increased G′ from 1200 Pa to 3300 Pa.

**Figure 4:**
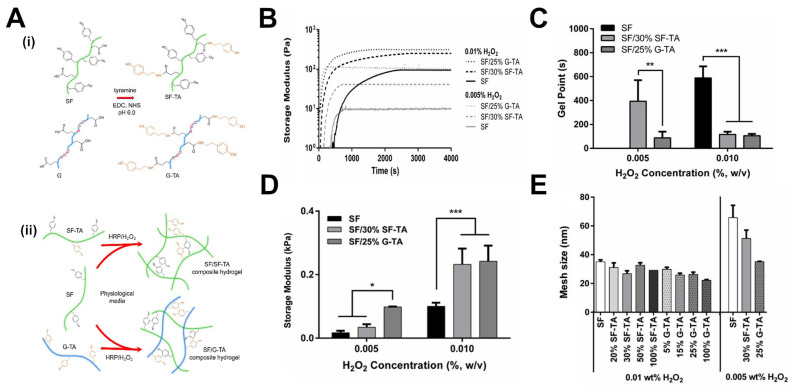
Enzymatic crosslinking strategy and its impact on hydrogel mechanical properties. (**A**) (**i**) Functionalization of silk fibroin (SF) and gelatin (G) with tyramine (TA) to generate phenol-containing precursors (SF-TA, G-TA); (**ii**) subsequent enzymatic crosslinking via horseradish peroxidase (HRP) and H_2_O_2_ leads to covalently bonded hydrogels. (**B**) Time sweep rheology showing gelation kinetics of 3% hydrogels with 0.005% and 0.01% H_2_O_2_. (**C**) Gelation time points of 3% (*w*/*v*) silk-based hydrogels varying H_2_O_2_ concentrations, determined from rheological time sweep analysis. (**D**) G′ at equilibrium for the same formulations (n = 3, * *p* < 0.05, ** *p* < 0.01, and *** *p* < 0.001). (**E**) Mesh sizes for SF and G formulations calculated by rubber elasticity theory are shown as a function of functionalized biopolymer composition and H_2_O_2_ concentration. Reproduced with permission from Hasturk et al., Biomaterials; published by Elsevier, 2020 [97].

Another widely investigated approach in the hydrogel literature is cell encapsulation using enzymatically crosslinked protein-based hydrogels. Recently, silk fibroin (SF), a natural structural protein of silkworm cocoons, and silk-gelatin (SF-G) hydrogels were functionalized with tyramines (TA) and crosslinked using horseradish peroxidase (HRP) and H_2_O_2_ [97]. This system enabled the formation of covalent dityrosine bonds between native tyrosine residues in addition to the non-native tyramines, where HRP catalyzes the oxidation of phenolic groups in the presence of H_2_O_2_, allowing reactive radicals to couple into stable crosslinks as presented in Figure 4A. This resulted in a covalent 3D hydrogel without an additional crosslinker. Unlike the previously discussed study that used stoichiometric control, this approach modulates H_2_O_2_ concentration to tune the crosslinking density and thereby control the mechanical behavior of the resulting hydrogels. Real-time rheometry showed that reducing the H_2_O_2_ concentration from 0.01% to 0.005% slowed the gelation process and resulted in lower storage moduli for all tested hydrogel formulations, as shown in Figure 4B,C. This delayed gelation and softening behavior is due to the reduced formation of reactive radicals at lower H_2_O_2_ concentrations, which limits the rate and extent of crosslinking. This approach yielded hydrogels with G′ ranging from ~250 Pa at 0.01% H_2_O_2_ to ~30 Pa at a lower concentration of 0.005% H_2_O_2_ as shown in Figure 4D.

**Figure 5:**
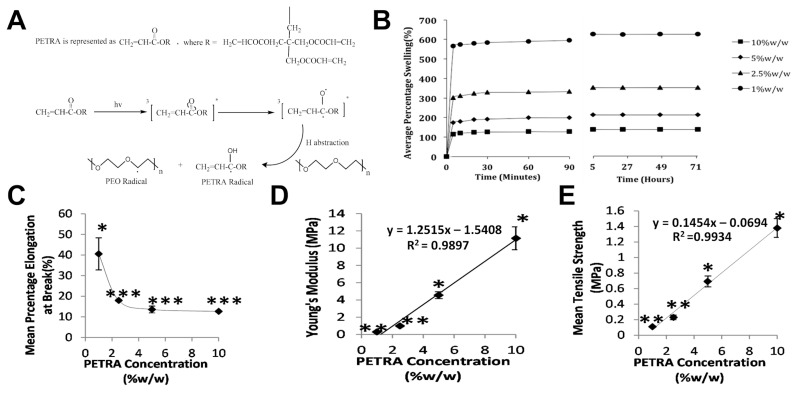
Effect of different PETRA concentrations (% *w*/*w*) on PEO hydrogels. (**A**) Representation of PEO radical generation using PETRA. (**B**) Swelling behavior over time. (**C**) Percentage elongation at break. (**D**) Young’s modulus. (**E**) Tensile strength (n = 3; * significant differences between all groups; ** significantly different from PETRA concentrations = 5% and 10% *w*/*w*; *** significantly different from PETRA concentration = 1% *w*/*w*; *p* < 0.05). Reproduced with permission from Wong et al., Pharmaceutics; published by MDPI, 2015 [93].

Wound healing and tissue regeneration have also been attractive targets for hydrogel innovations. PEG hydrogels have garnered particular interest in this area [98]. In one study utilizing this material, PEG crosslinked via UV irradiation in the presence of pentaerythritol tetra-acrylate (PETRA, a photo-reactive crosslinker) showed promise toward skin regeneration. This platform allowed for tunable, photo-induced crosslinking by adjusting the PETRA concentration from 1% to 10% (*w*/*w*) as depicted in Figure 5A. As demonstrated by Figure 5B,C, increasing PETRA concentration from 1% to 10% decreased the swelling ratio from ~600% to ~100% and the percentage elongation at break from ~40% to ~12%. This corresponds with Figure 5D,E, which demonstrate the effect of PETRA concentration on Young’s modulus and tensile strength. In totality, these results clearly demonstrate that increasing crosslink density corresponds directly to an increase in hydrogel modulus, a decrease in swelling ratio, and typically a shortening of gelation time. These results are in line with modeling expectations based on the Flory–Rehner theory and classical rubber elasticity [8]. Complementary diffusion and swelling models developed by Peppas et al. support the inverse relationship between increased crosslinking and hydrogel expansion [99,100]. A more recent framework proposed by Richbourg et al. builds upon these earlier models by including mesh heterogeneity, topological constraints and network defects present in real systems [98].

#### 4.1.2. Crosslinker Architecture Governs Mesh Size Variation in Hydrogel Networks

Mesh size is the average distance between adjacent crosslink junctions within a hydrogel network [101,102]. This parameter plays an important and is often underexplored role in determining the ability of solutes to diffuse through the matrix, particularly in systems designed for therapeutic delivery [103]. One of the most effective ways of varying mesh size is through modulating crosslinker design parameters, such as length, flexibility, functionality, and topology of their junctions. By varying the mesh size, it is possible to regulate the diffusion of bioactive molecules, modulate the interaction of encapsulated cells with their environment, and optimize the local presentation of signaling cues, making it an important design characteristic for applications in drug delivery, growth factor release, and scaffold-based tissue engineering [104,105].

Critically, material performance across these applications depends on the molecular design of the polymeric backbone used, since this directly influences structural parameters such as crosslinking density, functionality, branching, and network mesh size. Returning to the previously discussed work on TA-modified SF [97], altering the ratio of TA-functionalized biopolymers in the presence of H_2_O_2_, the researchers modeled changes in the mesh size using rubber elasticity theory, which estimates the average mesh size from rheologically measured G′. It was determined that the mesh size decreased with increasing TA content (a proxy for crosslinking sites). For example, Figure 4E demonstrates that at 0.01% H_2_O_2_ concentration, mesh sizes decreased with increasing crosslink density. Similarly, these mesh size numbers can be modulated by altering H_2_O_2_ concentration (Figure 4E): at 0.005% H_2_O_2_, the mesh size increased almost two-fold for pure SF hydrogels and 30% SF-TA formulations. This increase is attributed to the lower availability of H_2_O_2_ to activate the HRP enzyme, which reduces the degree of crosslinking and subsequently increases mesh size.

**Figure 6:**
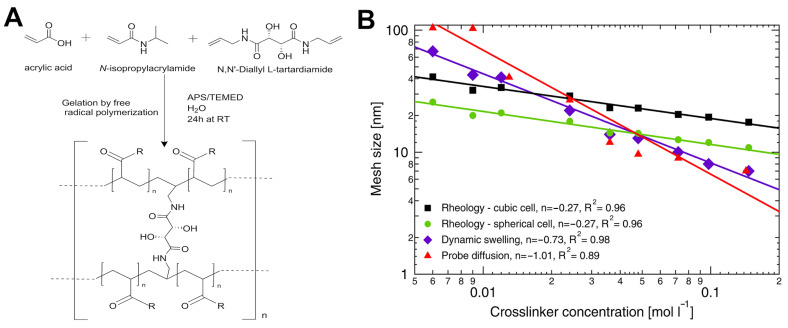
(**A**) Preparation scheme of poly(NIPAM-co-AAc) hydrogels. (**B**) Mesh size variation in poly(NIPAM) hydrogels as a function of crosslinker concentration (C_cl_), measured using three different methods: rheology (cubic and spherical models), dynamic swelling, and probe diffusion via PGSE NMR. Reproduced with permission from Wisniewska et al., Journal of Applied Polymer Science; published by John Wiley & Sons, 2018 [101].

In contrast to the enzymatic approach for cell encapsulation described earlier, mesh size can also be tuned via altering the structure and concentration of the crosslinking agents. An example of this strategy involved the use of poly(N-isopropylacrylamide-co-acrylic acid) (pNIPAm) hydrogels chemically crosslinked with N,N′-diallyltartramide (DAT), a small bifunctional molecule containing allyl groups [101]. In this system, the allyl groups of DAT form covalent bonds with the pNIPAm, creating a stable covalently crosslinked network, as depicted in Figure 6A. To measure the mesh size, three techniques were used: oscillatory rheology with two different models (cubic and spherical), dynamic swelling, and pulsed gradient spin–echo (PGSE) NMR with dextran probes. They modeled the relationship between crosslinker concentration (C_cl_) and the mesh size (ξ) via a power-law dependence: ξ ∝ (C_cl_)^n^ to determine the scaling exponent (n). Results presented in Figure 6B support the previously discussed inverse relationships with negative exponents for all three techniques, suggesting that increasing C_cl_ results in a smaller mesh size. For instance, both rheological models showed an exponent of −0.27. Increasing the concentration of the crosslinker from 0.01 mol/L to 0.1 mol/L significantly decreased calculated mesh size from ~33.9 nm to ~24 nm and ~30.2 nm to ~22.9 nm for cubic and spherical models, respectively. Similarly, by calculating the mesh size through swelling and PGSE NMR techniques, decreases in mesh sizes from ~44.7 nm to ~28.2 nm and ~63.1 nm to ~35.5 nm were observed.

**Figure 7:**
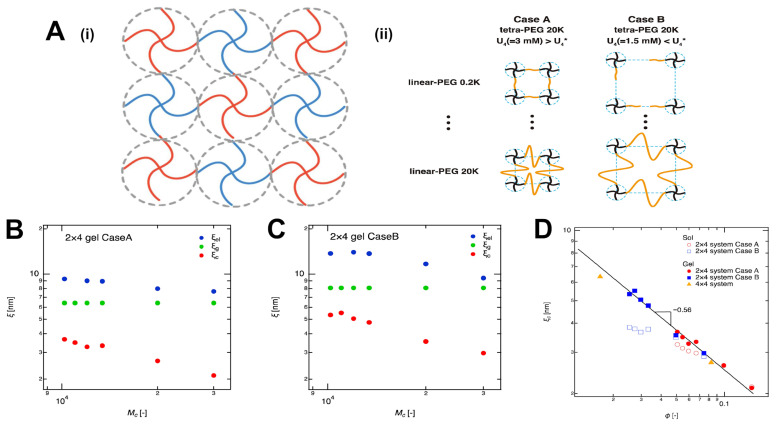
(**A**) Schematic representation of the different gel network design: (**i**) two tetra-PEG (4 × 4) network; (**ii**) tetra-functional (2 × 4) PEG network system with case A, representing a complete network, whereas case B represents an incomplete network. Blob size dependence on molecular weight between crosslinkers (Mc) for (**B**) case A and (**C**) case B. (**D**) Correlation blob size (ξ_c_) in function of total polymer volume fraction (φ). Reproduced with permission from Tsuji et al., Gels; published by MDPI, 2018 [106].

An analogous study was conducted on 2 × 4 and 4 × 4 PEG hydrogels, where the 2 × 4 configuration refers to a system where tetra-functional PEG macromers (four arms) are crosslinked using bifunctional linear PEG chains (two reactive ends) and the 4 × 4 configuration describes a network formed by linking two tetra-functional PEG components as depicted in Figure 7A [106]. To understand the structural variations at different scales, the study employed the “blob” framework, a conceptual model used to estimate mesh size through three types of characteristic lengths: the geometric blob (ξ_g_), calculated from theoretical crosslink spacing; the elastic blob (ξ_el_), derived from bulk shear modulus (G); and the correlation blob (ξ_c_), obtained via dynamic light scattering (DLS). Figure 7B,C showed that all three blob sizes decreased as the molecular weight between crosslinkers increased. For example, in case A, ξ_g_, ξ_el_, and ξ_c_ decreased from approximately 7.3 nm to 6.3 nm, 9.3 nm to 8.0 nm, and 3.9 nm to 2.3 nm, respectively. In case B, the decrease was not as pronounced: from ~8.5 nm to ~7.5 nm (ξ_g_), ~13 nm to ~9.5 nm (ξ_el_), and ~5.7 nm to ~4.2 nm (ξ_c_). Additionally, the researchers reported variation for ξ values in the correlation blob as a function of the polymer volume fraction (φ) and the configurations. Figure 7D shows that the mesh size for the 2 × 4 configuration varied from ~3.8 nm to ~2.2 nm and from ~5.5 nm to ~3 nm for cases A and B, respectively. The 4 × 4 configuration showed a similar significant decrease from ~6.3 nm to ~2.8 nm. These findings show that mesh size can be modulated through different approaches that involve polymer concentration, polymer functionalization, crosslinker concentration, and architecture design to obtain tunable sizes to meet specific application needs and architecture designs to obtain tunable sizes to meet specific application needs.

#### 4.1.3. Effect of Moisture Content on Hydrogel Mechanical Properties

Moisture content, often expressed as equilibrium water content or swelling ratio, plays a pivotal role in determining the mechanical behavior of hydrogels. Given that hydrogels are inherently water-swollen networks, variations in their hydration state directly influence key parameters such as modulus, toughness, and viscoelastic response. Generally, increasing moisture content (i.e., higher swelling ratio) leads to a reduction in the effective polymer volume fraction, thereby decreasing the hydrogel’s stiffness and tensile strength. This inverse relationship arises because water acts as a plasticizer, expanding the distance between polymer chains, reducing intermolecular interactions, and lowering the effective crosslink density per unit volume [107].

Studies on PEG and PVA-based hydrogels have consistently demonstrated this trend, showing that hydrogels equilibrated at higher relative humidities or when immersed in aqueous environments exhibit significantly lower moduli compared to their less swollen counterparts. For example, hydrogels with swelling ratios increasing from ~200% to ~800% can exhibit a two- to four-fold decrease in Young’s modulus, consistent with classical polymer network theories such as rubber elasticity and the Flory–Rehner model [8,9]. This effect is not solely limited to elasticity; moisture content also modulates time-dependent properties. Highly swollen hydrogels often display faster stress relaxation and greater creep under load due to increased chain mobility and reduced entanglement constraints.

Moreover, the interplay between moisture content and network architecture is critical. Hydrogels with side-chain crosslinking or short, rigid crosslinkers tend to retain water differently than those with flexible, long-chain crosslinkers, which can accommodate larger swelling without compromising network integrity. This suggests that the design of hydrogels for specific mechanical or biomedical applications must consider hydration-dependent behavior as an integral design parameter.

### 4.2. Viscoelastic Behavior

Viscoelasticity plays a crucial role in how hydrogels dissipate mechanical energy, a property essential for seamless integration with biological systems. By modulating crosslinker architecture, researchers can finely tune viscoelastic behavior through changes in network density and internal chain mobility. Incorporating dynamic bonds, such as reversible Schiff base linkages, further enables the material’s mechanical response to adapt under physiological conditions. This section explores how the structural design of crosslinkers governs key viscoelastic properties of hydrogels, including stress relaxation dynamics, half-stress relaxation time, and creep behavior.

#### 4.2.1. The Influence of Crosslink Architecture on Stress Relaxation Dynamics

Stress relaxation dynamics are essential mechanical traits of biological tissues, where a material subjected to constant strain undergoes a stress reduction via relaxation and redistribution of stress. These behaviors are governed by the tissue’s chemical composition, the constraints of the polymer chains within the network, and their spatial organization [108]. To mimic the behaviors of these biological systems, hydrogels have been designed with a variety of crosslinking architectures (as discussed in Section 2.2) [27].

In a recent study, three spatial organizations (SCX, LX, SX) were compared to determine how crosslinker architecture drives the viscoelastic properties of the resulting hydrogels. Investigators measured the stress relaxation dynamics using rheological indicators such as the G′, G″, and half-stress relaxation time (t_½_), which provide direct insight into how structural features translate into mechanical persistence over time [27]. The following Table 3 summarizes the findings:

Table 3 illustrates that the SCX structure maintains G′ values of approximately 103 Pa while exhibiting G″ values below 101 Pa. These data indicate the capacity of the SCX structure to store energy with low losses, demonstrating a strong resistance to stress decay and elastic behavior. The LX structure yields a slightly lower storage modulus, as expected from our discussion above on crosslinking architecture. However, in this case, the G″ presented increased to between 101 and 102 Pa, indicating a more prominent viscous response than the SCX structure. Finally, the SX network offers an intermediate mechanical response. Its centralized architecture produces G′ values ranging from 10^3^ to 10^4^ Pa, coupled with G″ around 102 Pa, striking a balance between elastic integrity and viscous dissipation. This design enables effective energy storage while permitting controlled stress relaxation.

The differences in viscoelastic behavior among SCX, LX, and SX architectures arise from crosslink distribution and chain mobility. SCX has pendant reactive groups along its backbone, promoting the formation of dense, highly crosslinked networks with limited chain mobility and minimal stress relaxation. In contrast, LX utilizes telechelic polymers with terminal functional groups, resulting in sparser, end-linked networks that exhibit greater segmental mobility and enhanced stress relaxation [109]. Finally, SX networks feature multiple arms around a core [110], yielding robust yet dynamic junctions that balance elasticity with controlled energy dissipation.

**Figure 8:**
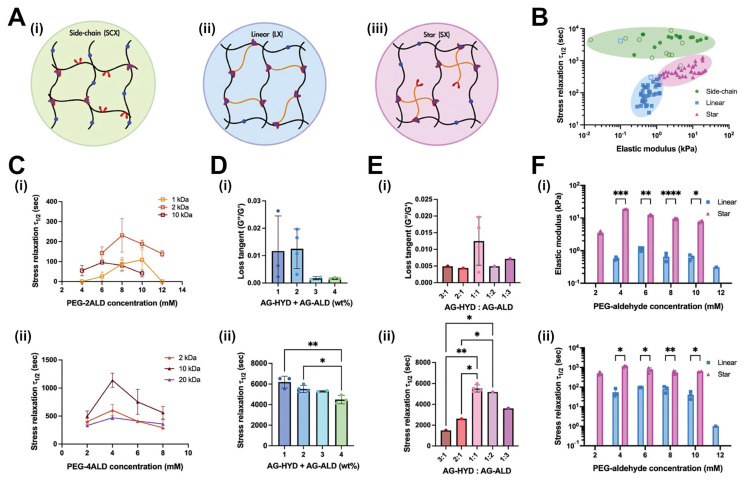
Rheological characterizations of SCX, LX, and SX hydrogels. (**A**) Schematic representation of side-chain crosslinker (SCX) (**i**), linear crosslinker (LX) (**ii**), and star crosslinker (SX) (**iii**) architectures. (**B**) The stress relaxation half-time and elastic modulus of hydrazone hydrogels with SCX, LX, and SX. (**C**) Stress relaxation half-time of LX (**i**) and SX (**ii**) hydrogels with varying linear polyethylene glycol-dialdehyde (PEG-2ALD) and four-arm polyethylene glycol-aldehyde (PEG-4ALD) cross-linker concentration of different MWs. (**D**) Loss tangent (**i**), and stress relaxation half-times (**ii**) of SCX hydrogels with varying total cross-linker concentration, maintaining an alginate–hydrazone (AG-HYD) to alginate–aldehyde (AG-ALD) ratio of 1:1. n = 3, 4, 2, and 4 replicates for 1, 2, 3, and 4 wt.%, respectively. Data are presented as mean ± SD. One-way ANOVA with Tukey’s post hoc test. * *p* ≤ 0.05, ** *p* ≤ 0.01. (**E**) Loss tangent (**i**), and stress relaxation half-time (**ii**) of hydrogels with varying AG-HYD to AG-ALD ratios, all at a total cross-linker concentration of 2 wt.%. n = 1, 1, 4, 1, and one replicate for 3:1, 2:1, 1:1, 1:2, and 1:3 ratios, respectively. Data are presented as mean ± SD. One-way ANOVA with Tukey’s post hoc test. * *p* ≤ 0.05, ** *p* ≤ 0.01. (**F**) Elastic modulus (**i**) and stress relaxation half-time (**ii**) of LX versus SX hydrogels. Data are presented as mean ± SD. Multiple unpaired Welch’s *t*-test with Holm-Šídák multiple comparison. * *p* ≤ 0.05, ** *p* ≤ 0.01, *** *p* ≤ 0.001, **** *p* ≤ 0.0001. Reproduced with permission from Lin et al., Advanced Healthcare Materials; published by John Wiley & Sons, 2024 [27].

This architectural influence is further evaluated in Figure 8B, which shows hydrogels from all three topologies within a 2D space defined by stress relaxation time and elastic modulus. Each crosslinking scheme forms a clear region in the parameter space with SCX hydrogels clustering toward the slower stress relaxation t_½_ (>10^3^ s), while LX hydrogels occupy an area of the space characterized by rapid relaxation and low stiffness. Finally, SX hydrogels represent a midpoint in network architecture, providing a controllable balance of elastic and viscous responses [27].

**Figure 9:**
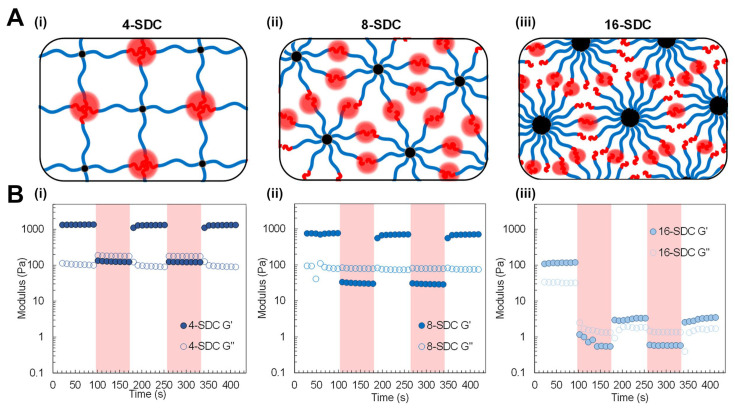
Graphical representation (**A**) of different physically assembled SDC hydrogel networks. (**B**) Rheological analysis of 4-SDC (**i**), 8-SDC (**ii**), and 16-SDC (**iii**) hydrogels using dynamic amplitude sweep at 5.0 wt % (γ = 0.1% for 240 s (white), then γ = 40% for 240 s (red), then γ = 0.1% for 240 s, then γ = 40% for 240 s, then γ = 0.1% for 240 s, all at ω = 1 rad/s). Reproduced with permission from Cosgrave et al., Biomacromolecules; published by American Chemical Society, 2024 [26].

A closer examination of this SX architecture reveals that variations in crosslinker functionality, even within the same topological class, can significantly alter the viscoelastic response. Hydrogels from 4, 8, and 16-arm star diblock copolypeptides (SDC) were produced under the same conditions (Figure 9A). The four-arm system recovered 99.4% of its initial storage modulus (G′ = 1330 → 1322 Pa) after exposure to extreme shear, indicating a highly elastic, resilient network with slow stress relaxation. The eight-arm system showed partial recovery (92.6% G′ = 760 → 704 Pa), suggesting greater chain mobility and moderately faster stress dissipation. Most notably, the 16-arm network exhibited a near-complete loss of mechanical stiffness, with G′ dropping from 120 Pa to just 3 Pa, achieving only 2.5% recovery, consistent with rapid stress relaxation and a highly dynamic, reconfigurable network [26].

These findings indicate that higher-arm SX crosslinkers promote faster stress relaxation by enhancing network reconfigurability. While increasing the number of arms introduces more connection points, it also promotes dynamic exchange and reduces network stability. At very high arm numbers (e.g., 16), the junctions become highly reconfigurable, leading to rapid stress relaxation and poor recovery, indicative of a soft, dissipative, and structurally unstable network [26].

In summary, these findings illustrate that both the spatial arrangement of crosslinkers and their molecular architecture govern how a hydrogel resists or dissipates stress over time. Pendant-based SCX networks enable slow relaxation through distributed anchoring, while flexible LX systems yield quickly due to their sparse connectivity. SX networks represent a tunable intermediate, though their mechanical performance is highly sensitive to crosslinker design. This highlights a strategic opportunity in materials engineering: by modulating network topology, crosslinker functionality, and spatial organization, one can tailor stress relaxation behavior to suit specific applications. Achieving such control is crucial for creating hydrogels that maintain mechanical integrity under cyclic loading or adapt to dynamic physiological environments.

#### 4.2.2. Half-Stress Relaxation Time (t_½_) as a Function of Crosslinker Structure

The half-stress relaxation time (t_½_) is defined as the duration it takes for a material to reduce its internal stress to half its original value following deformation [68]. It provides a practical method for evaluating the viscoelastic behavior of a hydrogel and facilitates direct comparison between various crosslinking architectures. Several experiments have shown that changing the architecture of the crosslinker can shift t_½_ across a wide timescale [27,62].

If a constant strain is applied at t = 0, and the resulting stress σ(t) relaxes over time, one can calculate t_½_ using Equation (2):

(2) $$\sigma \left(t_{½}\right)=\frac{1}{2}\sigma (0)$$

In a Maxwell model, stress decays exponentially:

(3) $$\sigma \left(t\right)=\sigma (0)e^{-t/\tau }$$

where τ is still the relaxation time defined in Section 3.2.2. Solving for $t_{½}$, we have Equation (4):

(4) $$\frac{1}{2}\sigma \left(0\right)=\sigma (0)e^{-t_{½}/\tau }$$

So, in general, for systems that can be modeled as simple Maxwell models:

(5) $$t_{½}=\tau \;ln\;ln\;\left(2\right)\approx 0.693\tau$$

One clear example of the influence of the LX, SX, and SCX architectures on t_½_ was provided by researchers, who measured the t_½_ from LX PEG-2ALD of varying molecular weights (1, 2, and 10 kDa), SX PEG-4ALD of comparable arm length (2, 10, and 20 kDa), and SCX AG-HYD to AG-ALD (1 to 4 wt.%) with stoichiometries of AG-HYD to AG-ALD of 3:1, 2:1, 1:1, 1:2, and 1:3. The results are reported in Figure 8C–E [27].

This figure illustrates how crosslinker architecture, molecular weight, and concentration influence the viscoelastic properties of hydrazone-crosslinked hydrogels, as measured by stress relaxation half-time (t_½_). In Figure 8C, PEG-2ALD (LX) and PEG-4ALD (SX) crosslinkers were evaluated across a range of molecular weights and concentrations. For both architectures, intermediate concentrations yielded the longest t_½_ values, with a clear dependence on molecular weight: higher MW PEGs (particularly 10 kDa) led to slower stress relaxation, likely due to increased network entanglement or reduced bond exchange dynamics. PEG-4ALD constructs exhibited significantly longer relaxation times overall, underscoring the impact of multivalent connectivity on mechanical persistence. In Figure 8D and E, bulk hydrogels formulated with AG-HYD and AG-ALD were evaluated by varying either the total polymer content (wt%) (Figure 8D(ii)) or the hydrazine-to-aldehyde ratio (Figure 8E(ii)). Increasing total polymer concentration led to shorter t_½_ values, suggesting that denser networks facilitate more rapid stress redistribution. Conversely, tuning the stoichiometry revealed that a 1:1 molar ratio of AG-HYD to AG-ALD produced the most persistent stress relaxation, while off-stoichiometric formulations (3:1 or 1:3) resulted in decreased t_½_, likely due to suboptimal crosslinking. Together, these results highlight multiple molecular design levers for fine-tuning hydrogel viscoelasticity.

**Figure 10:**
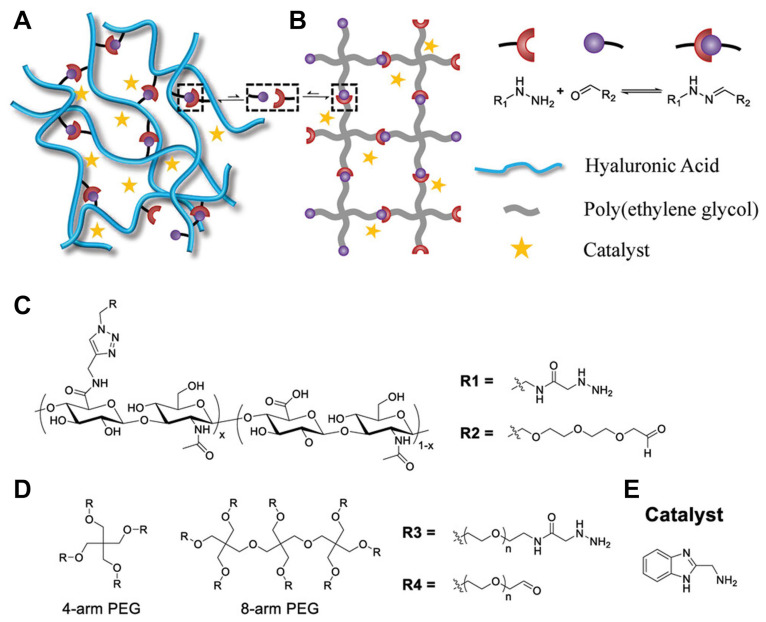
Schematic representation of two polymer network architectures: (**A**) crosslinking of linear polymers and (**B**) end-linking of star-shaped polymers. (**C**–**E**) Chemical structures of: (**C**) hydrazine and aldehyde functionalized HA, (**D**) hydrazine and aldehyde functionalized PEG, and (**E**) catalyst used to accelerate hydrazone exchange. Reproduced with permission from Lou et al., Advanced Materials; published by John Wiley & Sons, 2021 [62].

This same pattern was confirmed in another study where all hydrogels used the same dynamic hydrazone bonding but varied in crosslinker structure. SCX networks were again built from HA with pendant aldehyde groups (Figure 10A,C), while SX networks used PEG crosslinkers with either four or eight arms (Figure 10B,D). The SCX hydrogel had a terminal relaxation time (t_t_, used here as a proxy for t_½_) of ~16,000 s. The four-arm SX hydrogel was slightly faster, at ~11,500 s, while the eight-arm SX network showed the slowest relaxation, with t_t_ of ~48,000 s. These results indicate an apparent architectural influence: more arms and connections slow down the rate at which stress can be dispersed [62].

Both studies suggest that crosslinker architecture directly affects t_½_. LX networks relax quickly due to their simple linear configuration and fewer crosslinking points. In contrast, SX networks, especially those with more arms, introduce greater connectivity, resulting in slower relaxation. Finally, SCX networks tend to relax the most slowly. Understanding this hierarchy, SCX > SX > LX, provides a relevant design rule for tailoring hydrogels to meet specific mechanical needs, whether the goal is quick dissipation or sustained stress retention.

#### 4.2.3. Control of Creep Behavior Through Crosslinker Design

Creep behavior refers to the gradual deformation of a material caused by the application of a constant force overtime. In hydrogels, creep behavior is intricately linked to the design of their crosslinking networks [111].

In several investigations, researchers have studied the creep behavior of hydrogels as a function of their spatial crosslinking design. To indicate the long-term resistance of a material to deformation, rheological measurements, such as the loss tangent (G″/G′), which reflects the balance between viscous flow and elastic energy storage, and the before mentioned t_½_, which represents the duration over which internal stresses persist before dissipating. In Figure 8D(i),E(i), SCX networks showed loss of tangent values up to ~0.012. This indicates that the SCX network is highly elastic and has limited viscous rearrangement capability (G′ >> G″) due to its highly crosslinked network. This high rigidity was confirmed by measuring the t_½_ of the SCX structure, which presents values between 4000 and 6000 s. In contrast, the SX networks balanced rigidity and stress dissipation, with G′ values around 20 kPa (Figure 8F(i)), and t_½_ near 1000 s (Figure 8F(ii)). Finally, LX systems, which have a simpler structure relying on terminal linkages, showed stiffness less than 2 kPa (Figure 8F(i)) and t_½_ below 200 s (Figure 8F(ii)), signifying faster mechanical relaxation and increased susceptibility to long-term deformation [27].

**Figure 11:**
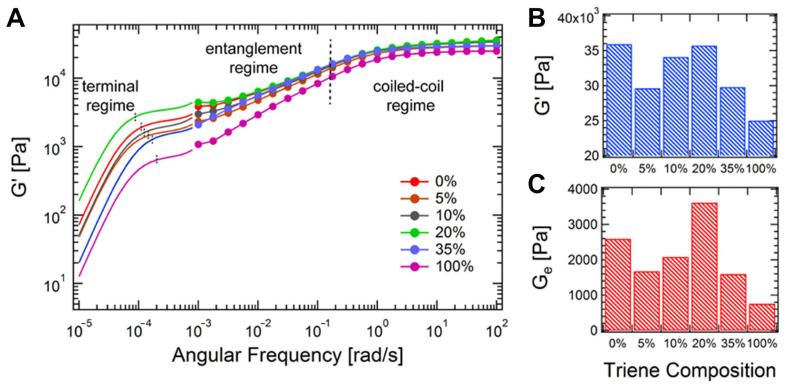
Comparison of the mechanical properties of branched hydrogels at different triene compositions. (**A**) Master curves at 25 °C from oscillatory shear and creep experiments; (**B**) comparison of the high-frequency moduli; (**C**) comparison of the entanglement moduli, determined from the converted low-frequency plateau. Reproduced with permission from Tang et al., Frontiers in Chemistry; published by Frontiers, 2014 [112].

A similar trend has been observed in protein-based hydrogels with tunable branching. In one system, dithiols create LX networks in protein-based hydrogels with adjustable branching. Simultaneously, trienes introduce varying branching, resulting in SX architecture. By increasing the triene ratio, the authors were able to modify network complexity and observe its effect on creep behavior (Figure 11). According to the findings, entirely linear systems (0% triene) exhibited a greater rubbery plateau modulus (G∞) of approximately 35 kPa and an entanglement modulus (Ge) of around 2.6 kPa, indicating superior physical entanglement and resistance to deformation. Conversely, fully branched networks (100% triene) reduced G∞ to approximately 25 kPa and Ge to around 900 Pa, reflecting diminished connectivity and increased deformability. This shows how small architectural changes greatly affect material performance under mechanical stress over time [112].

Notably, creep behavior is influenced by more than just the static network topology. A compelling example can be found in light-responsive hydrogels. In these systems, adjusting the light intensity modifies the mobility of dynamic crosslinks, enabling external control over relaxation behavior. In one example, as illumination increased, t_½_ dropped from 3300 to 1500 s, while the creep rate increased from 0.04 to over 0.11 MPa^−1^·s^−1^. These findings mirror what was observed with branching and network density: faster relaxation, induced by architecture or external cues, leads to greater time-dependent deformation. It serves as a reminder that the mechanical life of a hydrogel is not fixed at synthesis but can also be actively modulated post-fabrication in certain stimuli-responsive systems [113].

Creep can be modulated by engineering networks to minimize the loss of stored energy. Whether you use rigid architectures like SCX and LX or dynamic, light-responsive linkages, the goal is to tailor how the hydrogel relaxes and stresses over time. Mastering this balance lets you design hydrogels that start out as stiff as needed and, crucially, keep performing under sustained load in the complex conditions of real biological environments.

### 4.3. Engineering Mechanics in Hydrogels

Poor mechanical strength often limits the application of hydrogels [114]. To address this issue, several crosslinking-based strategies have been developed. Among these strategies are approaches that involve biomimetic reversible unfolding crosslinkers, double network architectures, hierarchical crosslinking, and multi-scale strategies that induce strain-stiffness. These methods increase hydrogel stiffness under deformation, mimicking biological materials.

#### 4.3.1. Biomimetic Design via Reversibly Unfolding Crosslinkers

Unfolding crosslinkers are dynamic chemical agents that are mechanically responsive to deformation or external forces and reversibly unfold representative systems including protein-based domains such as titin, streptavidin, and filamin, which show force-induced unfolding and refolding cycles [115]. In comparison with conventional covalent bonds, these reversible crosslinkers can dissipate energy and better adapt to the configuration of the network, even under stress, promoting toughness and mechanical adaptability [116]. Taking advantage of this characteristic, unfolding crosslinkers have been introduced in hydrogels to replicate the adaptive nonlinear stress response and mechanics of soft tissues [117]. One such approach embeds cryptic binding sites (hidden motifs that unfold under mechanical stress to create reversible bonds) within pNIPAAm hydrogels [117]. This system was designed to include folded loops joined by reversible cryptic bonds and dangling chains with reactive ends. Under thermal and mechanical stimuli, the loops unfold and reveal the cryptic sites, which can form reinforced crosslinks with the chains. For instance, the presence of cryptic bonds dramatically decreased hydrogel swelling in comparison with systems that lacked these cryptic sites. This behavior was replicated in a computational model under a dimensionless constant applied force of F = 3, in which the swelling response was reduced by ~3.2%, and ~50% for the lateral and transversal extension of the hydrogel volume.

**Figure 12:**
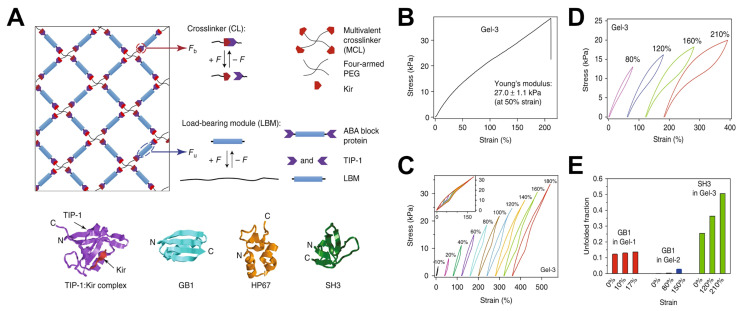
(**A**) Schematic of a hydrogel made from force-responsive crosslinkers and LBMs, including TIP-1:Kir, GB1, HP67, and SH3 domains. Mechanical performance of Gel-3 mediated by reversibly unfolding SH3 domains. (**B**) Stress–strain curve for Gel-3 under uniaxial tension. (**C**) Representative loading–unloading cycles for Gel-3 at increasing strain levels. (**D**) Theoretical modeling of Gel-3 stress–strain behavior under cyclic loading. (**E**) Fraction of unfolded SH3 domains increases with applied strain. Reproduced with permission from Wu et al., Nature Communications; published by Nature, 2018 [116].

A complementary design involves the integration of modular protein domains. In contrast with the hidden nature of cryptic sites discussed previously, these domains are independent units within a large protein that unfold and refold under applied forces [116]. Unlike the first approach, wherein a reinforcement structure was formed through secondary bonds and the compaction of its network, this latter design involves the use of dynamic domains to enhance the toughness and extensibility of the hydrogel. A classic example of this type of system, SH3 domains, has been used in load-bearing modules (LBMs) encapsulated within protein chains, while non-covalent TIP-ent TIP-1:Kir interactions served as reversible crosslinkers in the hydrogel network [116], as shown in Figure 12A. This domain was characterized by unfolding at low forces, ~25 pN, being able to dissipate mechanical energy during deformation, and refolding quickly after the release of stress, allowing the material to recover its mechanical integrity. Among the three different hydrogel formulations developed by the authors, Gel-3 was the only system in which the unfolding of SH3 enhanced the mechanical performance of the hydrogel. Figure 12B shows the marked improvement in extensibility observed, an increase of ~215%. Unlike the cryptic sites system, a relevant hysteresis upon multiple stretching–relaxation cycles from 10% to 180% strain was also observed (Figure 12C), confirming that the energy applied to the system was successfully dissipated through the unfolding of the protein as shown in Figure 12D,E.

**Figure 13:**
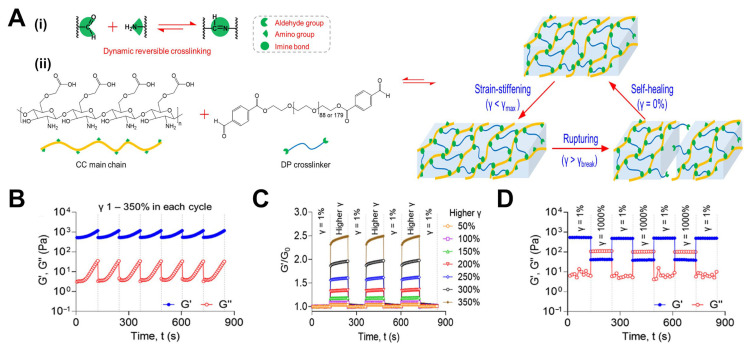
(**A**) Schematic of the dynamic imine-crosslinked hydrogel: (**i**) reversible imine bond formation and (**ii**) network illustration of biomimetic responses under mechanical strain. Rheological evaluation of CC/DP8k hydrogels under increasing deformation: (**B**) G′ and G″ during cyclic strain sweeps. (**C**) G′/G0 ratios at γ = 1%. (**D**) G′ and G″ under alternating cycles of low and extreme strain. Reproduced with permission from Liu et al., ACS Applied Materials and Interfaces; published by American Chemical Society, 2022 [118].

Another biomimetic strategy involves the use of dynamic covalent imine bonds as reversible crosslinkers [118]. Unlike the hidden nature of cryptic sites or unfolding motifs of modular proteins, this approach utilizes the dynamic exchange between dibenzaldehyde-terminated telechelic PEG (DP) and amine-functionalized PEG (CC) precursors with different chain lengths: DP4k and DP8k (4 and 8 kDa, respectively). Figure 13A(i,ii) shows that chemical gelation was induced to form a reversible imine hydrogel network capable of adapting to its environment in a strain-responsive manner. Rheological analysis with increasing deformation depicted in Figure 13B–D showed the DP8k system reached a viscoelastic regime near the breaking point, followed by immediate mechanical stiffness recovery close to 100% of the initial G′ after unloading, returning to G′ ≈ G_0_ ≈ 924 Pa, while maintaining a low G″ (~10–100 Pa) across cycles. This DP8k system outperformed the shorter DP4K crosslinkers, resisting fracture strains up to 790% and showing enthalpic stiffness behavior, which allowed for energy dissipation. These results demonstrate how a reversibly unfolding crosslinker can be used not only to successfully dissipate mechanical energy but also to adapt mechanically to the network architecture in response to external forces, enabling their utility in biomimetic designs.

#### 4.3.2. Double-Network Approaches with Complementary Crosslinker Architectures

**Figure 14:**
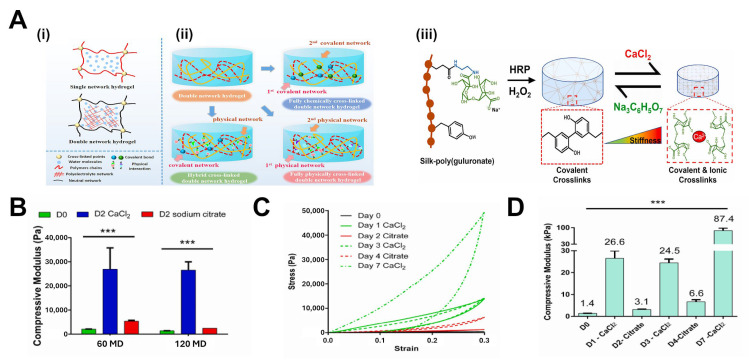
Theoretical and experimental validation of a double-network hydrogel system combining covalent and ionic crosslinking. (**A**) Schematic representation of network architectures: (**i**) single-network versus double-network hydrogel; (**ii**) classification of double-network hydrogels based on their crosslinking types, including chemically and physically crosslinked configurations. Reproduced with permission from Yin et al., Advances in Colloid and Interface Science; published by Elsevier, 2023 [121]. (**iii**) SF-PG double-network hydrogel. (**B**) Compressive modulus of SF–PG hydrogels made with 60 or 120 min MD silk fibroin (n = 5, *** *p* < 0.001). (**C**) Stress–strain curves for 120 min MD SF–PG hydrogels over seven days. (**D**) Quantified compressive modulus over seven days of cyclic treatment (n = 5, *** *p* < 0.001). Reproduced with permission from Hasturk et al., Polymer; published by Elsevier, 2023 [122].

DN hydrogels are an interpenetrating network (IPN) system that includes two independent polymer networks with complementary mechanical roles [119]. One of the networks commonly consists of a covalently crosslinked polymer network that provides a stable and elastic structure, while the other consists of reversible, physical interactions that allow energy dissipation, such as hydrogen bonding or ionic coordination, as is depicted in Figure 14A(i,ii) [120,121]. Ionic coordination is a widely used strategy due to its reversibility and tunability, which typically uses cations such as Cu^2+^, Ca^2+^, Al^3+^, Ba^2+^, Zn^2+^, Fe^2+^, and Fe^3+^ to physically crosslink polymer chains via electrostatic complexation [121].

The use of Ca^2+^ as a strategy to engineer DN systems has been recently studied in a silk–poly(guluronate) (SF–PG) hydrogel system [122]. Figure 14A(iii) represents this approach, where the first network is formed by the enzymatic covalent crosslinking of silk fibroin via HRP and H_2_O_2_, to form dityrosine bonds that provide the mechanical integrity of the hydrogel. Meanwhile, a second, ionic crosslinking occurs between the PG and Ca^2+^ ions, introducing reversible and stimuli-responsive behavior to the system. This system is treated with exogenous calcium chloride (CaCl_2_) to form these ionic linkages, followed by sodium citrate, which will disengage this secondary crosslinking. Upon treatment with CaCl_2_, the compressive modulus of 7.5% *w*/*v* SF-PG hydrogels increased from ~1.4 kPa to ~27 kPa. Subsequently, when the Ca^2+^ was sequestered by adding sodium citrate, the modulus decreased back to ~3.1 kPa. Figure 14B–D illustrates the changes in mechanics observed over a week, confirming the robustness and reversibility of the system.

**Figure 15:**
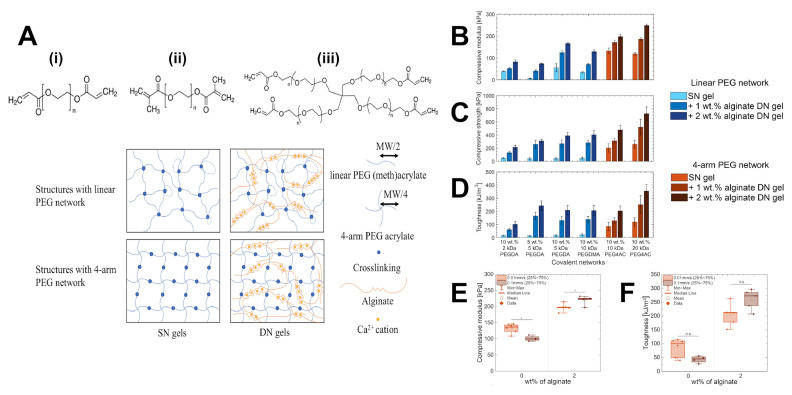
Structural design and mechanical reinforcement in PEG–alginate double-network (DN) hydrogels. (**A**(**i**–**iii**)) Chemical structures and network schematics for PEGDA, PEGDMA, and PEG4AC, along with representative architectures of SN and DN hydrogels formed with linear PEG (top) or four-arm PEG (bottom). Mechanical characterization comparing linear and four-arm PEG-based SN and DN systems: (**B**) compressive modulus, (**C**) compressive strength, and (**D**) toughness. Strain-rate sensitivity analysis for four-arm PEG-based hydrogels: (**E**) compressive modulus and (**F**) toughness at 0.01 mm/s and 0.1 mm/s (The label “n.s.” indicates that the statistical tests did not reveal a significant difference and “*” represents a significant difference with *p* < 0.05). Reproduced with permission from Huang et al., Acta Biomaterialia; published by Elsevier, 2022 [123].

Another recent application of Ca^2+^ coordination to form a DN hydrogel was developed in a PEG–alginate system [123], where the first network was formed via covalent crosslinking of PEG via thiol–ene Michael addition, using two different geometries: linear PEG and four-arm PEG (PEG4AC). The linear PEG consisted of two terminal functional groups, whereas the PEG4AC had a SX structure with four reactive sites. The second network consisted of alginate chains ionically crosslinked with Ca^2+^, as depicted in Figure 15A(i–iii). Through quasistatic compression testing, the authors evaluated the mechanical behavior of these hydrogels. Figure 15B demonstrates that the compressive modulus of the DN hydrogels were significantly higher than the single network alternatives (from ~60 kPa to ~160 kPa for the LX networks and ~120 kPa to ~270 kPa for the SX networks). These findings support the trend observed in the SF-PG system, unsurprisingly, a higher density of bonds increases the strength of the material. A similar trend was observed for compressive strength in Figure 15C, with SX systems were approximately 5-fold stiffer than LX counterparts. The DN system with 2 wt.% alginate reached values up to 700 kPa, decreased to 400 kPa for the LX variant. When evaluating toughness, the SX structure once again outperformed the LX, absorbing more energy per cubic meter—up to around 350 kJ/m^3^ compared to 260 kJ/m^3^ for the linear structure. This trend, shown in Figure 15D, was consistent for single network systems, where the LX architectures reached values of approximately 20 kPa, while the SX analogs reached up to 140 kPa. Furthermore, mechanical behavior at different strain rates was notably different. For example, for single network systems, the compressive modulus decreased from ~130 kPa at 0.01 mm/s to ~100 kPa at 0.1 mm/s. While in DN systems at 2 wt.% alginate, the modulus increased from ~200 kPa to ~230 kPa, as presented in Figure 15E, suggesting different strain-dependent mechanics. Similarly, Figure 15F shows that the toughness of the single network hydrogels decreased from ~100 kJ/m^3^ to 50 kJ/m^3^, while DN hydrogels increased from ~220 to ~275 kJ/m^3^.

In an analogous study, a fibrin hydrogel formed an IPN with a disulfide-crosslinked HA [124]. Unlike the previous systems that used synthetic building blocks, this study included entirely bioderived materials. The hydrogel was fabricated through the polymerization of fibrin using thrombin and Ca^2+^, while HA was chemically crosslinked through a thiol–disulfide exchange reaction between HA-SH and HA–SSPy (2-pyridyl disulfide) derivatives. This enabled a supporting behavior where fibrin provided fibrous structure, while HA offered high water retention and mechanical stabilization, preventing network compaction and enhancing durability. Mechanical testing was performed, and it was found that the 2% fibrin-1% HA system achieved G′ values up to ~2500 Pa after swelling, which represented an improvement of almost two-fold in comparison with only 2% fibrin gels, G′~1300 Pa. This fibrin IPN system prevented hydrogel compaction and delayed the degradation of the fibrin. These findings illustrate the tunability of DN hydrogels via complementary covalent and ionic crosslinking networks, providing a mechanically adaptable and dynamic system useful for diverse biomedical applications, such as tissue engineering, biomimetic models, and regenerative medicine.

#### 4.3.3. Enhancing Mechanical Performance Through Hierarchical Crosslinking

Hierarchical crosslinking refers to the integration of various types of interactions within a single network [125]. This hierarchical design approach mimics natural systems like those present in the native ECM, facilitating multiscale connectivity that enhances strength and durability [126,127,128].

One example of chemical hierarchical design is the incorporation of both weak and strong bonding sites within the same polymer network. This is exemplified in a study that designed hydrogels using N,N-dimethylacrylamide (DMAA), acrylic acid (AAc), and UPyEA. This molecule contains UPy, which can form strong and reversible hydrogen bonds with another UPy unit. Consequently, the hydrogel structure includes both covalent bonds (provided by DMAA and AAc) as well as hydrogen bonds in the UPy units. This creates a hierarchy in which weak bonds absorb energy and break, while strong bonds maintain the shape of the network [128].

By maintaining a 50:50 molar ratio between DMAA and AAc, while varying the concentration of UPyEA between four molar ratios: 0, 0.2, 0.4, and 0.8 (see Table 4) a significant range of properties was achievable. These hydrogels were tested by a tensile test to study the influence of this hierarchical design on mechanical performance.

Table 4 enables us to understand the impact of hydrogen bond density. As the amount of UPyEA increased, the modulus rose from ~75 to ~1200 kPa. Additionally, the maximum stretch increased from ~3100% to ~4300%, indicating that the material became significantly stiffer, but also much more extensible.

This enhancement arises from the interplay between the dual bonding strategy within the network. The weak bonds break initially, allowing energy dissipation, while the stronger bonds hold the material together.

## 5. Biological and Biomedical Applications

Hydrogels with tunable and optimized mechanical and viscoelastic properties through crosslinker architecture design are of particular relevance to biomedical applications. These applications tend to require specific material properties, which may vary substantially depending on the application of interest. This section examines how the crosslinker architectures discussed so far can be designed to translate into tunable mechanical and viscoelastic properties, and how these are applied in biological and biomedical environments. See Table 5 for a complete summary of biological and biomedical applications discussed.

### 5.1. Cell-Material Interactions

Cells are able to sense and interact with their environment, leading to different cellular outcomes [136]. These interactions are mediated through mechanosensors such as integrins [137,138], which respond to signals like topography, porosity, adhesion motifs, and hydrogel matrix stiffness or viscoelasticity [139]. By tuning crosslinker architecture, these cues can be precisely tuned to influence cellular behavior [139].

**Figure 16:**
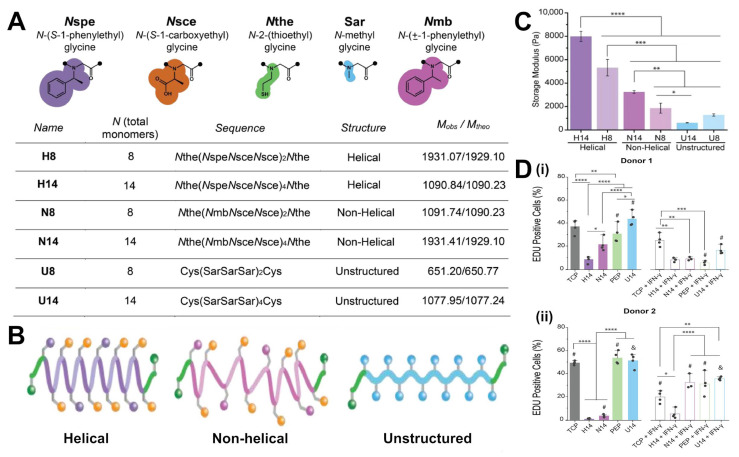
(**A**) Chemical specification for each peptoid crosslinker. (**B**) Peptoid-crosslinkers structures used as an architecture design to modulate bulk hydrogel mechanics. (**C**) Storage moduli via oscillatory rheometry for different hydrogel formulations. (**D**(**i**,**ii**)). Proliferation quantified for different hydrogel formulations (* denotes *p* < 0.05, ** *p* < 0.01, *** *p* < 0.001, **** *p* < 0.0001. All statistics were calculated by one-way ANOVA with post-hoc Tukey HSD test. & indicates *p* < 0.01 and # indicates *p* < 0.0001 between that condition with and without IFN-γ). Reproduced with permission from Morton et al., Acta Biomaterialia; published by Elsevier, 2023 [38].

One important caveat with classical hydrogel systems is that a variety of parameters are inherently coupled via crosslinking density, architecture, etc. Recently, attempts to decouple these properties, has led to the development of a variety of different hydrogel formulations, wherein one property (usually hydrogel stiffness) is tuned via an alternate, independent pathway. A prime example of this was recent work utilizing a variety of peptoid crosslinkers [37,38,39,140]. Through just minor side chain differences (and the resulting differences in secondary structure—see Figure 16A,B) stiffness was able to be modulated over a significant range. The HA hydrogels fabricated ranged from G′~0.6 kPa for the softest formulations (unstructured) to ~8 kPa for the stiffest formulations (helical), without modifying network connectivity, as shown in Figure 16C. Figure 16D(i,ii) shows that this stiffness difference was relevant to hMSCs seeded onto the hydrogel surface. The softer hydrogels enhanced the proliferation of the hMSCs by several fold, while also improving their immunosuppressive capacity.

A complementary strategy to enhance hMSCs adhesion, spreading, and metabolic activity was developed by varying the ratio of SF–tyramine and gelatine–tyramine in enzymatically crosslinked SF/gelatin hydrogels [97]. Through tuning crosslinking density, the authors were able to control stiffness and mesh size. Softer hydrogels with lower crosslinking density and larger mesh size promoted greater cell spreading, adhesion and viability. Overall, these studies illustrate that crosslinker architecture can directly influence hMSCs immunomodulation, proliferation, adhesion, and viability.

### 5.2. Therapeutic Delivery Systems

Therapeutic delivery systems refer to the targeted transport and release of drugs or cells in a controlled manner. The optimization of these systems is important for enhancing drug efficacy and administration for prolonged times and minimizing side effects, such as enzymatic degradation [141,142]. By modulating crosslinker design, these hydrogel systems can modify their mesh size, degradation kinetics, and stimuli responsiveness [143]. This section explores injectable formulations and stimuli-responsive networks for targeted delivery.

DN hydrogels are an excellent candidate for sustained release due to their mechanical stability, tunable porosity, and responsiveness to external stimuli [52]. Recently, alginate-templated silk and silk–gelatin hydrogel microbeads were utilized toward this end [129]. The system combined enzymatic crosslinking of SF-TA and G-TA via HRP/H_2_O_2_ with ionic alginate crosslinking, enhancing mechanical stability and allowing on-demand degradation. Encapsulated hMSCs and neural progenitor cells (NPCs) showed high viability and proliferation after mechanical extrusion and maintained ~70–80% viability under adverse conditions, such as acidic pH, TNF-α (as a proinflammatory challenge), UV exposure, and mechanical shear. In contrast with unencapsulated cells which showed lower viability (below ~50%). This finding supports DNs as promising injectable delivery platforms.

Drug delivery is a complementary approach in therapeutic delivery platforms. To achieve pH-responsive and controlled drug release, a pectin/PVA-based hydrogel was developed to deliver ceftriaxone, a drug to treat bacterial infections. This platform used 3-aminopropyl (diethoxy)methylsilane (3-APDEMS) as a bifunctional crosslinker to form covalent bonds [130]. By varying the crosslinker concentration from 0 to 20%, the authors modulated porosity (61–79%), swelling (300–1275%), and degradation rates to fine-tune drug diffusion. In physiological conditions, over 90% of ceftriaxone was released in 180 min, which demonstrated an efficient sustained delivery.

In parallel, PVA/carrageenan (a natural food additive derived from red seaweed) hydrogels were synthesized using (3-aminopropyl)triethoxysilane (APTES) as a crosslinker, enabling the creation of a dual chemically crosslinked injectable network [131]. The platform was pH-responsive and provided controlled swelling and a sustained release of cephradine, another antibiotic drug. This system demonstrated controlled release over multiple hours, with ~85% of the cephradine released in 7.5 h in simulated intestinal fluid (SIF). Crosslinker concentration modulated swelling behavior, with values ranging from 20% to 200%, which was the primary driver in drug diffusion. These studies highlight how crosslinker design parameters are crucial to modulating key parameters in injectable, stimuli-responsive and therapeutic delivery platforms.

### 5.3. Regenerative Medicine

Regenerative medicine aims to regenerate cells, organs, and tissue structures by promoting repair mechanisms or cues to restore normal functionality in the human body or introducing engineered substitutes [95,144]. This broad field includes subareas focused on tissue engineering, wound healing, and cell-based therapies, which use biomaterials that can mimic the dynamic properties of the ECM [145,146,147]. Crosslinker design plays a critical role in tuning hydrogel properties such as stiffness, viscoelasticity, biodegradation, and immunoactivation [43,148]. These hydrogel properties then impact cellular behavior, tissue integration, and scaffold remodeling. This section explores how different crosslinker architectures are applied in various regenerative medicine applications.

DN hydrogels have once again shown promise in this field due to their chemical and mechanical stability and tunable swelling properties [135]. Commonly, these DN hydrogels are used for tissue engineering applications to fabricate biomimetic scaffolds. One such application involved the implantation of a dual-crosslinked hydrogel scaffold to promote bone regeneration in a rat defect model with bacterial infection [132]. The system combined methacryloyl-modified silk fibroin (SilMA), methacrylated gelatin nanoparticles (MA-GNPs), and copper-doped bioglass (CuBGs), to form a UV-curable network with mechanical reinforcement and self-healing capacity. Once implanted in the defect site, the scaffold promoted structural regeneration by increasing bone volume fraction from ~35% to ~60% at two and four weeks, respectively.

Another design to engineer biomimetic tissues was an HA-based hydrogel system with variable stiffness and adhesivity, which aimed to replicate the ECM environment of the aortic valve [133]. In the design, the authors modified HA molecular weight and crosslinking density to modulate hydrogel stiffness, while methacrylated gelatin (GelMA) provided RGD motifs for cell adhesion. Encapsulated aortic valve interstitial cells (VICs) showed stiffness-dependent behavior, where softer hydrogels significantly increased glycosaminoglycan (GAG) production, a key component for valve ECM regeneration. In contrast, stiffer hydrogels promoted a myofibroblastic phenotype with high α-smooth muscle actin (α-SMA) expression, which is an indicator of undesirable matrix contraction and pathological remodeling.

A complementary strategy involved the use of an injectable hydrogel to support osteogenesis and immunomodulation in vivo by enzymatically crosslinking alginate–tyramine with sericin, which is a photoluminescent protein useful to control injectable hydrogels [134]. Graphene oxide was incorporated into the system to improve the osteogenic differentiation of rat bone marrow stem cells, while sericin promoted macrophage M2 polarization via NF-kB MAPK pathways. This M2 phenotype is associated with a pro-regenerative secretory profile, such as IL-10 and Arg-1 expression, promoting tissue repair and producing a more osteogenic environment. The authors achieved structural stability and gradual degradation of the system through implementing HRP/H_2_O_2_ crosslinking.

Similar systems have been used to target wound healing and skin regeneration applications. As a strategy to support these processes, a 3D-bioprintable HA-based hydrogel was developed using a double-crosslinked system composed of methacrylated HA (HA-MA) and thiolated HA (HA-SH) [135]. The 3D-bioprintable scaffold exhibited swelling ratios up to ~95%, which promoted water uptake and debridement, supporting and enhancing wound healing and tissue regeneration. The system also demonstrated reliable enzymatic degradation rates (~90% over 11 days), which is associated with angiogenesis, a key process in tissue repair. Additionally, the system enabled the sustained release of Nafcillin, a drug used to prevent bacterial infection during healing. These examples demonstrate how crosslinker design impacts cell fate, immune response, and scaffold performance in regenerative medicine applications.

## 6. Future Perspective and Challenges

### 6.1. Emerging Crosslinker Technologies

To address the mechanical constraints of traditional crosslinking networks, researchers are investigating alternative approaches that employ bioinspired, responsive, and programmable systems. These innovative strategies seek to improve stiffness and stress relaxation properties over classical systems.

A promising approach for developing crosslinkers is provided by mimicking mechanisms found in nature, such as coiled-coil protein self-assembly, which enables the formation of reversible yet stable networks. For instance, the reversible binding observed in mussel byssal threads is replicated through the coordination of histidine to Zn^2+^. This dynamic interaction forms strong, non-covalent bonds that stiffen the hydrogel network when Zn^2+^ is present and relax it when absent, allowing precise control over viscoelastic behavior and energy dissipation [149]. Owing to their versatility, these materials can emulate soft tissues, such as articular cartilage, which need to be both elastic and energy-dissipative to perform under cyclic loading [150].

Molecular modeling has emerged as a valuable tool for describing and predicting how crosslinking architecture influences the mechanical performance of polymer networks. By modeling and predicting structure–property relationships, these models can optimize the design of crosslinker strategies tailored to specific mechanical requirements. A recent computational study applied a coarse-grained molecular dynamics simulation to demonstrate that variations in network configuration (single or interpenetrating networks) significantly impact the stress–strain behavior and energy dissipation of dynamically crosslinked hydrogels. In this study, the authors found that interpenetrating networks improved bond formation and the overall mechanical resilience of the system compared to single networks. This behavior was attributed to the distribution and mobility of non-covalent crosslinkers within the network, which were identified as key factors influencing the mechanical strength of the system.

Another promising direction involves “smart” crosslinkers that respond to external stimuli such as pH, temperature, or light [151]. These stimuli enable the hydrogel to change its structure, leading to processes like swelling, shrinking, or sol–gel transitions in response to specific environmental conditions [152]. This built-in adaptability allows the network to alter its behavior under physiological or mechanical stressors, which is especially advantageous for applications like drug delivery or pressure-responsive systems [152,153,154].

Finally, switchable crosslinkers enable the development of programmable hydrogel systems. For example, one approach employs permanent covalent bonds, which provide mechanical stability, and reversible physical interactions, such as host–guest complexes with light responsive spiropyran, which allow real-time adjustments using light. These dual-crosslinked hydrogels exhibit high strength, energy dissipation, and behavior that can be controlled with UV and visible light [155]. This configuration enables the materials to react dynamically and reversibly, making them well-suited for applications such as wearable sensors [156].

These new technologies indicate a transformation in crosslinker design, moving from thinking of them only as passive connectors to active elements that allow hydrogels to respond, adapt, and function like living tissues.

### 6.2. Challenges in Crosslinker Design

Despite significant progress in hydrogel crosslinker innovation, several challenges impede their efficacy and broader adoption. These challenges involve balancing mechanical properties and biocompatibility, scaling intricate crosslinking techniques, and resolving ongoing issues with clinical applications.

A significant challenge in developing crosslinkers is balancing mechanical strength and biocompatibility. Since crosslinkers that improve stiffness and durability often use synthetic materials, these can reduce cytocompatibility [157]. In a recent study, researchers proposed a solution by creating a hydrogel made of 2-methacryloyloxyethyl phosphorylcholine (MPC). They crosslinked it with N,N′-methylenebisacrylamide (MB) using the cationic initiator, 2,2′-azobis-[2-(1-3-dimethyl-4,5-dihydro-1H-imidazol-3-ium-2-yl)] propane triflate (ADIP), resulting in high deformability and strong adhesion while maintaining low cytotoxicity. The authors demonstrated that reducing crosslinking density by adjusting monomer–initiator interactions was essential for achieving flexibility and cell compatibility [158].

Simultaneously, scalability presents a significant obstacle, especially with complex multi-component crosslinkers. Many sophisticated systems function well in lab environments, but their synthetic procedures or purification methods often prove challenging to replicate on an industrial scale [159]. For example, a study proposed a scalable method for creating elastic gelatin-based hydrogel films using waterborne polyurethane as a crosslinker. This approach maintained excellent mechanical strength and strain recovery while utilizing cost-efficient and reproducible synthesis methods [160]. It emphasizes the importance of ensuring manufacturing compatibility when designing smart materials.

Finally, translating hydrogels with encapsulated cells into clinical applications involves more than just optimizing materials [161]. A recent investigation into cartilage repair using GelMA-based hydrogels revealed that factors such as cell source, isolation protocol, expansion duration, and delivery method greatly influence results. While the procedure encouraged in vivo regeneration, the authors emphasized the importance of standardizing every step to guarantee reproducibility and alignment with surgical processes [159]. These issues pose significant challenges for clinical translation [159].

Overall, tackling these challenges necessitates improved chemistry, more innovative architecture, and integrated strategies that concurrently consider manufacturing, evaluation, and clinical needs. Only then will we unlock the full potential of the crosslinker architecture design space.

### 6.3. Future Research Directions

As hydrogel technology progresses, research must explore crosslinker architecture as a flexible, responsive component to meet complex biological needs. This evolution requires new strategies combining spatial and temporal control, data-driven optimization, programmable material properties, and tailored therapies.

A promising direction in crosslinker design is the use of spatiotemporally programmed mechanical environments. A recent study introduced macroporous hydrogels reinforced with stiff protein fiber-based crosslinkers, which provided localized mechanical cues, while the surrounding matrix gradually degrades. This synergy directed stem cell differentiation and improved bone regeneration in vivo. It demonstrated that the interplay between spatial stiffness and regulated degradation, enabled by tailored crosslinker structures, promotes early osteogenesis and subsequent tissue remodeling by offering mechanical support for cellular commitment and facilitating infiltration and vascularization during the healing process [162].

Simultaneously, machine learning shows great promise by accelerating crosslinker optimization. Conventional trial-and-error approaches can be time-consuming and resource-intensive, particularly when numerous formulation parameters must be altered simultaneously. A recent study utilized supervised learning models to predict droplet volumes considering viscosity, nozzle size, pressure, and cell concentration. This machine learning strategy allowed for swift optimization with high accuracy, surpassing manual adjustments and minimizing material waste [163]. The findings highlight how data-driven models can advance our understanding of crosslinker design rules and improve bioprinting precision, resulting in more efficacious and consistent systems.

Finally, tailoring crosslink mechanics to align with the unique properties of patient tissues is essential for the progress of personalized medicine. A recent study of hydrogel-based soft bioelectronics demonstrated that advanced network designs and crosslinker processing methods enable precise stiffness adjustments, accommodating various tissues, from soft brain (~1 kPa) to rigid tendon (~1 GPa). This mechanical flexibility ensures enhanced conformity and effectiveness in wearable and implantable devices, highlighting the significance of crosslinking systems designed to address individual anatomical and therapeutic needs [163].

Together, these emerging research directions highlight the growing need for structurally supportive crosslinkers that offer intelligence, adaptability, and customization. As these ideas develop, they will shape the next generation of hydrogel systems used in regenerative medicine, diagnostics, and beyond.

## Figures and Tables

**Table 1:** All crosslinking techniques discussed, along with their key features, advantages, and limitations.

Crosslinker Type	Hydrogel Systems	Range of Mechanical and Viscoelastic Properties	Key Features	Advantages	Limitations	Reference
Synthetic	PEGDA, PEG-(multiarm), PVA, MBAA, PAMAM dendrimers	PVA/PEG-Tensile strength: 2.93–4.41 MPa, Elongation at break: ~450–573%, Compressive tangent modulus: 0–4.6 MPa at strain from 10–60%	Biocompatible, tunable mechanics, chemically versatile, often inert	Predictable structure, facile functionalization, customizable network mechanics	Lacks bioactivity, requires bioactive cues	[20,27,28,29,30]
Peptide/Protein	MMP-cleavable peptides, avidin-biotin, coiled-coils, GelMA	GelMA—Compressive modulus: 20–300 kPa, Young’s modulus: 27.1–114.4 kPa, Stress–strain: 0–15 kPa at strain from 0–20%	Biological origin, enzyme-degradable, responsive, cell interactive	Cell-responsive degradation/remodeling, bioactive	Less mechanically robust, susceptible to proteolysis	[31,32,33,34,35]
Peptoid/ Peptidomimetic	Helical peptoids, D-peptides, foldamers	Helical peptoid—Compressive modulus: ~2–7 kPa, Storage modulus: ~0.5–2.3 kPa	Sequence-defined, protease-resistant, some structural control	High stability, tunable stiffness, long-term integrity	Synthetic complexity, cost	[36,37,38,39,40]
Polysaccharide-Based	Alginate, HA, Chitosan, Dextran	Alginate-GelMa—Storage modulus: ~5–50 Pa, Loss modulus: ~10–350 Pa, Compressive modulus: ~25–280 kPa	Natural, hydrophilic, often ionically/covalently modified	Biocompatibility, bioactivity, mild gelation	Mechanical weakness, reversible (ionic) bonding	[3,41,42,43,44,45,46,47]
Hybrid/Composite	PEG-peptide, silica nanoparticles, nanoclays	Self-assembling peptide—Storage modulus: 14.43–452,400 Pa, Loss modulus: 2.569–80,680 Pa, Yield stress: 0.32–7012 Pa	Combines synthetic and biological elements	Synergistic properties, biofunctionality + robustness	Complex design, potential heterogeneity	[48,49,50]
Linear	PEGDA, diamines, MBAA	PEG-2ALD—Storage modulus: Young’s modulus: 3–11 kPa, >1000 Pa, Loss modulus: ~10–100 Pa, Stress relaxation half time: 1–100 s	Difunctional, forms chain-end linkages	Simple, homogeneous networks possible	Difficulty inducing gelation	[20,28,30,51]
Star/Branched	Multi-arm PEG, streptavidin	4-arm PEG—Storage modulus: ~0–15,000 Pa, Loss modulus: ~1–5000 Pa	Multivalent, increased connectivity	Higher stiffness, more robust networks	May lead to loss of homogeneity	[20,27,33]
Side-Chain Crosslinking	GelMA, HAMA	AG-HYD—Young’s modulus: 1–17 kPa, Storage modulus: ~1000 Pa, Loss modulus: <1 Pa, Stress relaxation half time: 4000–6000 s	Crosslinking along backbone	Stiff networks, higher crosslink density	Loop formation, brittle behavior	[24,27,35]
Dendritic	PAMAM dendrimers, hyperbranched polyesters, polyglycerols	PAMAM—Storage modulus: ~0.2–31 kPa, Adhesive strength: 25–29 kPa	Tree-like architecture with high terminal group density, very high functionality	Extremely high crosslink density, rapid gelation, tunable surfaces for biofunctionality or stimuli-responsiveness	Synthetic complexity, potential loss of homogeneity, cost, intra-particle looping	[30]

**Table 2:** An overview of crosslinking mechanisms and chemistries.

Crosslinking Mechanism and Sub-Type	Bond Nature	Typical Gelation Kinetics/ Network Uniformity	Signature Mechanical or Dynamic Behaviors	Key Advantages and Representative Uses	Main Limitations/ Design Caveats	Reference
Chain-growth photopolymerization	Covalent (radical polymerization)	Seconds to minutes, can outrun diffusion	Elastic solid, initial high stiffness, microgels can act as defects	Rapid spatiotemporal control, light-patterning for 3D culture, in situ curing	O_2_ inhibition, residual sol fraction, can be brittle	[54,55,56]
Step-growth photopolymerization	Covalent (radical, step-growth)	Seconds, uniform conversion, oxygen-tolerant	More homogeneous modulus, good toughness	Fast, cytocompatible, bio-inks, microfluidics	Requires photoinitiator and light access, stoichiometry critical	[54]
SPAAC click	Covalent (azide + cyclooctyne)	Minutes, catalyst-free, uniform	Elastic, can be degradable	Injectable, bio-orthogonal in vivo gels	High cost, limited light control unless photo-uncage used	[57]
Michael-type additions	Covalent (base-catalyzed)	Tunable: seconds to hours, homogeneous if mixed well	Elastic, very cell-friendly	No radicals, no UV, easy bio-functionalization during gelation	Competing mono-thiol motifs lower crosslink density, pH-dependent	[58,59,60,61]
Kinetic-tuning (catalyzed hydrazone, etc.)	Same as parent bond	Catalyst decouples gel time from final modulus	Allows tunable injectability vs. modulus	Optimize delivery vs. defect density	Extra catalyst must be biocompatible or cleared	[59,62]
Dynamic covalent: imine/hydrazone/oxime	Reversible covalent	Tunable exchange rate (pH controlled)	Stress-relaxing, self-healing, viscoelastic	Cell-responsive matrices, injectable shear-thinning gels	Hydrolytic drift shifts equilibrium, slower at neutral pH for imines	[63,64]
Dynamic covalent: disulfide, Diels–Alder, boronate ester	Reversible covalent	Redox- or T-dependent, from minutes to hours	Tunable τ, shape-memory or thermo-morphing	Injectable depots, on-demand remolding	Requires stimulus for re-flow, can creep under load	[65,66,67]
Hydrogen-bonded networks	Non-covalent (H-bonds)	Rapid, but reversibility across wide timescales	Tough, strain–stiffening, pronounced stress relaxation	Self-healing, temperature-responsive	Weak under high humidity/heat, hysteresis on cycling	[68,69,70]
Host–guest inclusion	Non-covalent (inclusion complex)	Fast association, dissociation usually μs to s	Shear-thinning, quick self-recovery	3D printing, injectable therapeutics	Affinity set by host/guest choice	[71,72]
Metal–ligand coordination	Non-covalent (coordination)	Very fast, pH/ionic-strength controlled	Sacrificial bond toughness, viscoelastic	Tough DN gels, wound adhesives, pH-switchable systems	Chelators or pH shifts dissolve network, metal toxicity must be managed	[73,74,75,76]
Hybrid/multi-network	Often covalent and non-covalent	Determined by fastest chemistry	High modulus and high toughness, multi-phase relaxation	Load-bearing soft tissues, stretchable sensors	More complex synthesis, balance of network fractions is critical	[51,77,78]

**Table 3:** Storage and loss modulus of hydrogels with SCX, LX, and SX over 3600 s. Hydrogel formulations: SCX gel, 2 wt.% alginate with AG-HYD to AG-ALD ratio of 1:1; LX gel, 2 wt.% AG-HYD, 8 mm PEG-2ALD (2 kDa); and SX gel, 2 wt.% AG-HYD, 4 mm PEG-4ALD (10 kDa) [27].

Properties	SCX	LX	SX
Storage modulus—G′ (Pa)	~10^3^	<10^3^	10^3^ ~ 10^4^
Loss modulus—G″ (Pa)	<10^1^	10^1^ ~ 10^2^	~10^2^

**Table 4:** Physical properties of various hydrogels [128].

No.	Molar Ratio DMMA/AAc/UPyEA	Modulus (kPa)	Max Stress (kPa)	Strain at Break	Strain Energy Density (kJ m^−3^)
1	50/50/0	74 ± 10	74 ± 5	3150 ± 240	2370 ± 140
2	50/50/0.2	283 ± 18	87 ± 11	3850 ± 230	3230 ± 370
3	50/50/0.4	409 ± 36	132 ±17	3900 ± 280	4330 ± 320
4	50/50/0.8	1252 ± 108	234 ± 16	4340 ± 320	7160 ± 670

**Table 5:** Summary of biological and biomedical applications of crosslinker design strategies in hydrogels.

Biological and Biomedical Application	Hydrogel Type and Crosslinking Strategy	Key Outcomes	Reference
Immunomodulation of hMSCs	HA hydrogels crosslinked with peptoids; mechanical tuning without altering network connectivity	G′ range ~0.6–8 kPa; softer hydrogels (~0.6–3.2 kPa) increased proliferation (44–54%), IDO expression, circularity	[38]
hMSC adhesion and viability	SF-TA and Gel-TA enzymatically crosslinked (HRP/H_2_O_2_); crosslinking density modified	Softer hydrogels with lower density promoted spreading and viability; stiffer ones reduced adhesion and metabolic activity	[97]
Injectable cytoprotective delivery of hMSCs and NPCs	DN microbeads: SF-TA, Gel-TA (HRP/H_2_O_2_) + ionic alginate crosslinking	Higher viability after extrusion, ~70–80% under adverse conditions; unencapsulated cells dropped below ~50%	[129]
pH-responsive drug release of ceftriaxone	Pectin/PVA hydrogel crosslinked with 3-APDEMS; physical and chemical bonds	Porosity 61–79%, swelling 300–1275%, >90% drug release at 180 min	[130]
Sustained cephradine delivery	PVA/carrageenan hydrogel crosslinked with APTES; dual chemical and physical network	~85% release in 7.5 h; swelling 20–200%; release tuned by crosslinker content and pH	[131]
Bone regeneration in infected defects	DN hydrogel: SilMA, MA-GNPs, CuBGs; UV-curable, self-healing IPN	Bone volume increased from ~35% to ~60% between 2 and 4 weeks post-implantation	[132]
Aortic valve ECM mimicry	HA hydrogel with variable stiffness (via HA MW and crosslinking density) + Me-Gel for RGD motifs	Softer hydrogels increase GAG; stiffer hydrogels increase α-SMA (fibroblast vs. myofibroblast phenotype)	[133]
Bone regeneration and immunomodulation	Injectable alginate–tyramine hydrogel with sericin and GO; HRP/H_2_O_2_ crosslinking	M2 polarization increase IL-10, Arg-1; osteogenesis enhanced; scaffold degradable and injectable	[134]
Wound healing and skin regeneration	3D-bioprintable HA-based hydrogel (HA-MA + HA-SH); dual crosslinking (thiol–click + UV)	Swelling ~95%, degradation ~90% in 11 days, sustained Nafcillin release for infection control	[135]

## Data Availability

No new data were created or analyzed in this study. Data sharing does not apply to this article.

## Abbreviations

3-APDEMS	3-aminopropyl (diethoxy)methylsilane
AAc	Acrylic acid
ADIP	2,2′-azobis-[2-(1-3-dimethyl-4,5-dihydro-1H-imidazol-3-ium-2-yl)] propane triflate
AG-ALD	Alginate-aldehyde
AG-HYD	Alginate-hydrazone
APTES	3-aminopropyl)triethoxysilane
Arg-1	Arginase-1
BCN	Bicyclononyne
C_cl_	Crosslinker concentration
CaCl2	Calcium Chloride
CANs	Covalent adaptable networks
CB	Cucurbituril
CB[8]	Cucurbit[8]uril
CD-Ad	Cyclodextrin–adamantane
CD44	Cluster of differentiation 44
CuBGs	Copper-doped bioglass
DAT	N,N′-diallyltartramide
DMAA	N,N-dimethylacrylamide
DN	Double network
ECM	Extracellular matrix
EDTA	Ethylenediaminetetraacetic acid
EGDMA	Ethylene glycol dimethacrylate
ELP	Elastin-like protein
f	Average functionality of the crosslinkers
G	Bulk shear modulus
G-TA	Gelatin-tyramine
G_0_	Initial shear modulus
GAG	Glycosaminoglycan
GB1	Immunoglobulin-binding domain B1 of protein G
Ge	Entanglement modulus
GelMA	Gelatin-methacrylate
G′	Storage modulus
G″	Loss modulus
G″/G′	Loss tangent
G∞	Rubbery plateau modulus
H_2_O_2_	Hydrogen peroxide
HA	Hyaluronic acid
HA-MA	Methacrylated hyaluronic acid
HA-SH	Thiolated hyaluronic acid
HA-SSPy	Pyridyl disulfide-functionalized hyaluronic acid
hMSCs	Human mesenchymal stem cells
HP67	Folding protein 67 domain
HRP	Horseradish peroxidase
IDO	Indoleamine 2,3-dioxygenase
IL10	Interleukin-10
IPN	Interpenetrating network
k	Rate at which crosslinks can rearrange
k_eq_	Equilibrium constant
k_off_	Kinetics of bond breakage
k_on_	Kinetics of bond re-formation
Kir	Inwardly rectifying potassium channel
LAP	Lithium acylphosphinate
LBMs	Load-bearing modules
LX	Linear crosslinkers
M2	M2 macrophage phenotype
MA-GNPs	Methacrylated gelatin nanoparticles
MAPK	Mitogen-activated protein kinase
MB	N,N′-methylenebisacrylamide
MBAA	N,N′-methylenebis(acrylamide)
Mc	Molecular weight between crosslinkers
MMP	Matrix metalloproteinase
MPC	2-methacryloyloxyethyl phosphorylcholine
MSCs	Mesenchymal stem cells
MSNs	Mesoporous silica nanoparticles
n	Scaling exponent
NF-kB	Nuclear factor kappa-light-chain-enhancer of activated B cells
NMR	Nuclear magnetic resonance
NPCs	Neural progenitor cells
$\rho_{c}$	Critical extent of reaction at the gel point
PAMAM	Poly(amidoamine)
PAMPS	Poly(2-acrylamido-2-methylpropanesulfonic acid)
PEG	Polyethylene glycol
PEG-2ALD	Linear polyethylene glycol-dialdehyde
PEG-4ALD	4-arm polyethylene glycol-aldehyde
PEG-VS	Polyethylene glycol-vinyl sulfone
PEG4AC	4-arm polyethylene glycol
PEGDA	Polyethylene glycol diacrylate
PEGDMA	Poly(ethylene glycol) dimethacrylate
PETRA	Pentaerythritol tetra-acrylate
PGSE	Pulsed gradient spin–echo
pH	Potential of hydrogen
pHEMA	Poly(2-hydroxymethyl methacrylate)
pNIPAM	Poly(N-isopropylacrylamide-co-acrylic acid)
PVA	Poly(vinyl alcohol)
RGD	Arginine-Glycine-Aspartic acid
SCX	Side-chain crosslinkers
SD	Standard deviation
SDC	Star diblock copolypeptides
SF	Silk fibroin
SF-G	Silk-gelatin
SF-PG	Silk-poly(guluronate)
SF-TA	Silk fibroin-tyramine
SH3	Src Homology 3 domain
SIF	Intestinal fluid
SilMA	Methacryloyl-modified silk fibroin
SPAAC	Strain-promoted azide-alkyne cycloaddition
SX	Star crosslinkers
t_1/2_	Half-stress relaxation time
t_t_	Terminal relaxation time
TA	Tyramine
TIP	Tax-interacting protein
TIP-1	Tax-interacting protein 1
TNF-α	Tumor necrosis factor alpha
Tr	Relaxation time
UPy	Ureido-pyrimidinone
UPyEA	Ureido-pyrimidinone ethyl acrylate
UV	Ultraviolet
VICs	Valve interstitial cells
α-SMA	α-smooth muscle actin
γ	Strain
ξ	Mesh size
ξ_c_	Correlation blob
ξ_el_	Elastic blob
ξ_g_	Geometric blob
σ(t)	Resulting stress
τ	Relaxation time
φ	Polymer volume fraction
ω	Frecuency
